# Network Toxicology and Molecular Docking to Investigate the Non-AChE Mechanisms of Organophosphate-Induced Neurodevelopmental Toxicity

**DOI:** 10.3390/toxics11080710

**Published:** 2023-08-17

**Authors:** Juliana Alves da Costa Ribeiro Souza, Terezinha Souza, Isadora Louise Alves da Costa Ribeiro Quintans, Davi Farias

**Affiliations:** 1 Postgraduate Program in Bioactive Natural and Synthetic Products, Federal University of Paraíba, João Pessoa 58051-970, Brazil; julianaacrs@ltf.upb.br; 2 Laboratory for Risk Assessment of Novel Technologies, Department of Molecular Biology, Federal University of Paraiba, João Pessoa 58051-900, Brazil; terezinhamsouza@gmail.com; 3 Biosciences Department, Federal Rural University of Semi-Arid, Mossoró 59625-900, Brazil; isadoralouise@ufersa.edu.br

**Keywords:** aquatic contamination, ecotoxicology, environmental contamination, enrichment analysis, network biology, neurodevelopmental disorders, neurotoxicity of pesticides

## Abstract

Organophosphate pesticides (OPs) are toxic substances that contaminate aquatic environments, interfere with the development of the nervous system, and induce Neurodevelopmental Toxicity (NDT) in animals and humans. The canonical mechanism of OP neurotoxicity involves the inhibition of acetylcholinesterase (AChE), but other mechanisms non-AChE are also involved and not fully understood. We used network toxicology and molecular docking to identify molecular targets and toxicity mechanisms common to OPs. Targets related to diazinon-oxon, chlorpyrifos oxon, and paraoxon OPs were predicted using the Swiss Target Prediction and PharmMapper databases. Targets related to NDT were compiled from GeneCards and OMIM databases. In order to construct the protein–protein interaction (PPI) network, the common targets between OPs and NDT were imported into the STRING. Network topological analyses identified EGFR, MET, HSP90AA1, and SRC as hub nodes common to the three OPs. Using the Reactome pathway and gene ontology, we found that signal transduction, axon guidance, cellular responses to stress, and glutamatergic signaling activation play key roles in OP-induced NDT.

## 1. Introduction

The widespread use of organophosphate pesticides (OPs) in agriculture still raises concerns about their ecological impact and human health implication [1]. Surface waters and sediments can be contaminated by OPs from rainfall, runoff, improper disposal, or leaching from groundwater. This contamination can affect a wide range of aquatic organisms, such as fish, invertebrates, and algae, as well as non-target terrestrial organisms [2].

There are numerous toxic effects associated with OPs in fish, including hematological disturbances, gill, kidney, and liver alterations [3], oxidative stress, immune disorders, alteration of the intestinal microbiota [4], and behavioral disorders including a reduced predator escape response [5]. In humans, exposure to OPs during pregnancy and postnatal periods increases the risk of autism spectrum disorder [6], impaired IQ, and verbal comprehension [7].

Exposure to OPs poses a high risk of neurotoxicity, especially for the developing nervous system. The effects of this type of exposure can include cognitive, motor, and memory impairments, as well as changes in brain morphology, referred to as Neurodevelopmental Toxicity (NDT) [8].

Acute exposure to high doses of OPs, such as self-poisoning, can significantly inhibit the activity of the acetylcholinesterase (AChE) enzyme. As a result, acetylcholine accumulates in the synaptic cleft, and cholinergic receptors in the nervous system and neuromuscular junction are overactivated, leading to a cholinergic crisis. The symptoms of human poisoning include respiratory depression, cardiovascular complications, gastrointestinal problems, and mental confusion, and the mortality rate is 15–30% [9].

When environmental contamination occurs, OPs concentrations are typically low and may not significantly inhibit AChE activity. However, these low concentrations can still cause behavioral alterations and neurological damage, particularly during developmental stages [10]. Therefore, it is important to emphasize that non-AChE mechanisms are also involved in the NDT of OPs [8]. These non-AChE mechanisms may include glutamatergic excitotoxicity, apoptosis [11], disturbances in intracellular Ca^2+^ homeostasis, neuronal activity [12], the modulation of BDNF expression, and effects on the GABAergic and serotonergic systems [13].

Even though AChE inhibition is a common mechanism of action for OPs, there is evidence that the non-AChE effects can vary among different OP types. For instance, chlorpyrifos (CP) and malathion exhibited contrasting effects on zebrafish larvae. While malathion treatment led to morphological abnormalities in the brains of larvae, no such effect was observed with CP. Furthermore, CP-exposed larvae exhibited decreased swimming speed and increased resting time, whereas malathion-exposed larvae showed increased swimming speed and decreased resting time [14].

A comparative analysis of seven OPs (acephate, CP, dichlorvos, diazinon, malathion, parathion, and profenofos) in planarians revealed shared toxicological endpoints among the OPs (except for acephate and parathion), such as abnormal body forms, increased viscosity, writhing behavior, and alterations in swimming speed during exposure to blue light. However, other toxicological endpoints differed between the OPs [15].

This study was motivated by the following question: Do OPs share non-AChE mechanisms that contribute to NDT? This information can be valuable in enhancing toxicity testing protocols, establishing more precise safety margins, and providing targets for more effective treatments in cases of exposure to these compounds.

Computational tools can enhance toxicity studies by providing fast and comprehensive results. Network toxicology, a branch of network pharmacology, is one of these tools.

Network pharmacology, an emerging approach in the pharmaceutical industry, allows the analysis of molecular and protein interactions, as well as the mechanisms of action, prediction of therapeutic targets and signaling pathways in drug development [16]. However, the applications of these methodologies have expanded beyond pharmaceutical research, encompassing diverse fields such as ecotoxicology, where network toxicology can be employed to assess the impact of exposure to environmental contaminants. For example, Iida and Takemoto (2018) performed gene alteration predictions related to human diseases to assess the risk of exposure to seven categories of environmental contaminants [17]. Sohrabi et al. (2020) utilized similar approaches to predict key regulatory genes associated with hepatotoxicity that were common among various pesticides [18]

Combining network toxicology and molecular docking can reveal interactions between the investigated compounds and their predicted targets, thereby helping to determine their toxicological properties [19]. Thus, network toxicology emerges as a powerful tool for evaluating exposure to environmental pollutants, providing a greater understanding of the effects on biological systems.

The aim of this study was to predict non-AChE targets that are shared among OP pesticides that contribute to NDT using network toxicology and molecular docking. 

## 2. Materials and Methods

### 2.1. Chemical Structures of OPs

**Figure 1:**
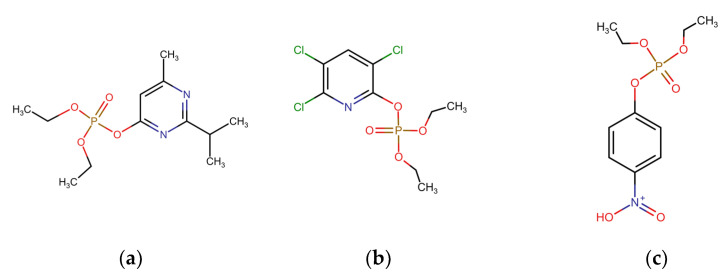
Chemical structure of oxon metabolites: (**a**) diazinon-oxon (CAS 962-58-3), (**b**) chlorpyrifos oxon (CAS 5598-15-2) and (**c**) paraoxon (CAS 311-45-5).

The 2D structures of the oxon metabolites of the OP pesticides diazinon (DZ), CP, and parathion were retrieved from the PubChem database (https://pubchem.ncbi.nlm.nih.gov) accessed on 28 February 2023. We chose the oxon metabolites over of the original compounds due to their direct association with NDT [20,21]. The images of the chemical structures of the organophosphates were generated using Marvin JS [22] and are shown in Figure 1.

### 2.2. Acquisition of OP Targets

The 2D chemical structures of paraoxon (PO), chlorpyrifos oxon (CPO), and diazinon-oxon (DO) were submitted to the Swiss Target Prediction database (http://www.swisstargetprediction.ch) accessed on 28 February 2023 and PharmMapper (http://www.lilab-ecust.cn/pharmmapper/) accessed on 28 February 2023 to predict target genes. We then entered the predicted target genes into the UniProt database (https://www.uniprot.org/) accessed on 28 February 2023 to retrieve the standard gene names, and duplicate entries were eliminated.

### 2.3. Prediction of NDT Targets

The GeneCards database (https://www.genecards.org/) accessed on 28 February 2023 and Online Mendelian Inheritance in Man (OMIM, http://omim.org/) accessed on 28 February 2023 were used to predict potential NDT targets. In February 2023, the related targets were collected using the keywords “neurodevelopmental abnormalities”, “neurotoxicity”, and “neurodevelopmental disorders”, along with the *Homo sapiens* species. The UniProt database was utilized to retrieve the standard gene names, and after merging the predicted targets for the three keywords from both databases, duplicate entries were removed.

### 2.4. Venn Analysis and Construction of Protein–Protein Interaction (PPI) Network

In order to identify the intersection genes as potential targets of each OP related to NDT, a Venn diagram was created by using Draw Venn Diagram tool (http://bioinformatics.psb.ugent.be/webtools/Venn/) accessed on 28 February 2023.

Protein–protein interaction (PPI) networks were constructed using the STRING database (https://string-db.org/) accessed on 28 February 2023. A minimum score of 0.7 was set, indicating a high level of confidence, and only experimental data were selected as the source of active interaction. The PPI network analyzes direct interactions between the inputted proteins and generates a network of interactions where proteins are represented as nodes and the interactions between them are represented as edges.

### 2.5. Topological Analysis of PPI Networks

The PPI networks were imported into Cytoscape 3.9.1. The cytoHubba plugin was utilized to compute the top 10 hub nodes using the Maximal Clique Centrality (MCC) method. The Analyze Network tool was used for topological analyses of the network, including degree (k), betweenness centrality (BC), clustering coefficient, closeness centrality (CC), and average shortest pathway (ASPL).

The measure k represents the number of connections a node has with other nodes in the network, i.e., it quantifies the number of edges that connect to it. Nodes with a high k value are important for information transmission in the network as they have more control over the flow of information. On the other hand, BC is a measure that indicates a node’s ability to connect other nodes in the network. A high BC value indicates that the node is important for communication between different parts of the network. In turn, the CC measure indicates how many direct neighbors of a node are interconnected, and a high CC value for a node indicates its involvement in a strongly connected cluster. Lastly, the ASPL measure indicates the average shortest path length between a node and all other nodes in the network [23].

### 2.6. Enrichment Analysis

An enrichment analysis was performed using the Consensus PathDB database (http://cpdb.molgen.mpg.de/) accessed on 28 February 2023 to explore the Gene Ontology (GO) and Reactome Pathway. The Biological Process (BP), Molecular Function (MF), and Cellular Component (CC) categories of GO were chosen. GO terms and Reactome Pathways with a *p*-value < 0.01 were considered statistically significant. The top 15 GO terms for BP, MF, and CC, as well as the top 30 Reactome pathways, were selected for each OP. Additionally, terms common to all three OPs that were directly related to the Nervous System superpathway, as classified by Reactome.org, were examined. GO and Reactome pathway graphs were generated with the ggplot2 package in RStudio.

### 2.7. Molecular Docking

A molecular docking analysis was conducted to explore the potential interactions between OPs and their hub nodes. The analysis followed the following steps:

(a) Macromolecule preparation: The 3D structures of the proteins were obtained from the PDB database (https://www.rcsb.org/) accessed on 28 February 2023 in PDB format. Discovery Studio 2021 was used to remove water molecules and ligands, add polar hydrogen bonds, and obtain the x, y, z coordinates for constructing the grid box. Energy minimization was performed using Swiss-PDB Viewer 4.1.0 software.
(b) Ligand preparation: The 3D structures of the ligands were retrieved from the PubChem database (https://pubchem.ncbi.nlm.nih.gov/) accessed on 28 February 2023 in SDF format and converted to PDB format using Open Babel.
(c) Docking simulation was performed using Autodock Vina [24], using a grid box with dimensions of 25 × 25 × 25 Å. The xyz coordinates obtained from Discovery Studio were used to select the center of mass for each macromolecule (Table 1). The poses were selected based on the lowest root-mean-square deviation (RMSD) values, with a maximum threshold of 2.0 Å.
(d) The analysis of the binding and generation of images was carried out using Discovery Studio 2021. The distance criterion between ligands and amino acid residues was established as <3.3 Å for hydrogen bonds [25] and <6.0 Å for π-π, π-alkyl and π-sigma interactions [26].

## 3. Results

### 3.1. Candidate Targets for OP-Induced NDT

After removing the duplicates, 388 targets were found for DZO, 343 for CPO, and 387 for PO in the Swiss Target Prediction and PharmMapper databases. Additionally, 3060 NDT-related targets were obtained from the Gene Cards and OMIM databases after removing the duplicates. Using a Venn diagram to identify the intersections of targets for each OP and NDT, we found 187, 121, and 181 targets for DZO, CPO, and PO, respectively. These targets were used to construct a PPI network using the STRING database, and the data were imported into Cytoscape for visualization and analysis of the hub nodes.

**Figure 2:**
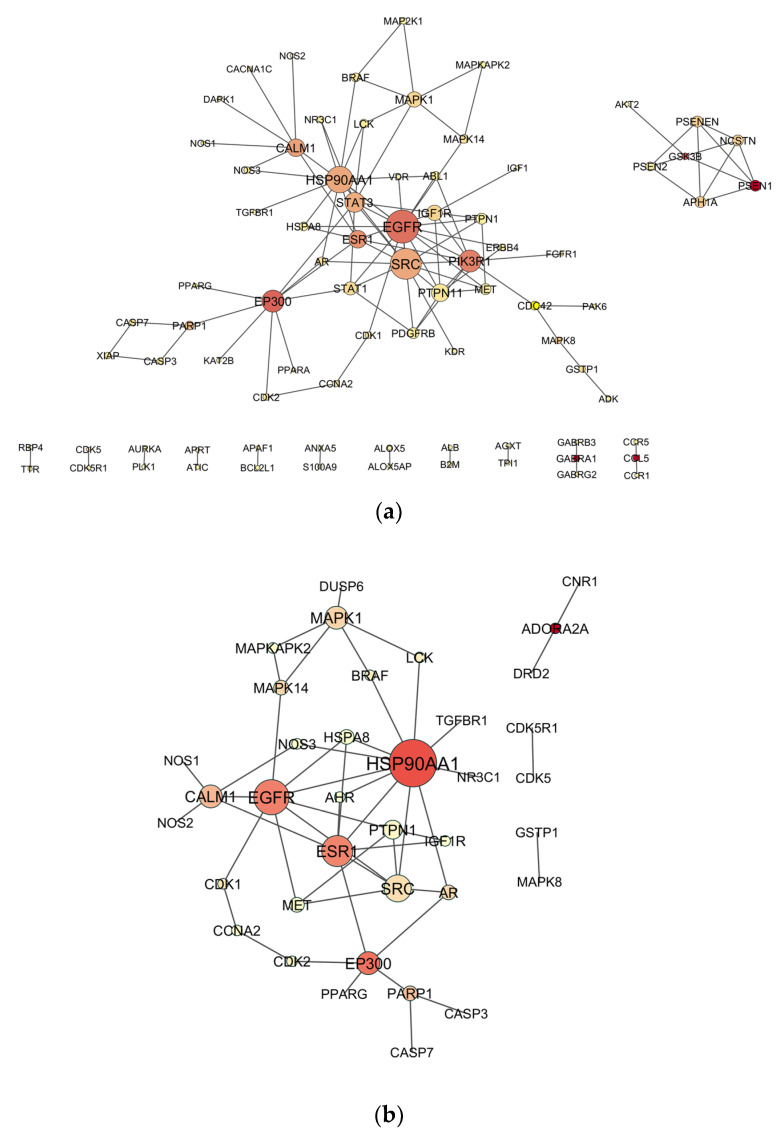

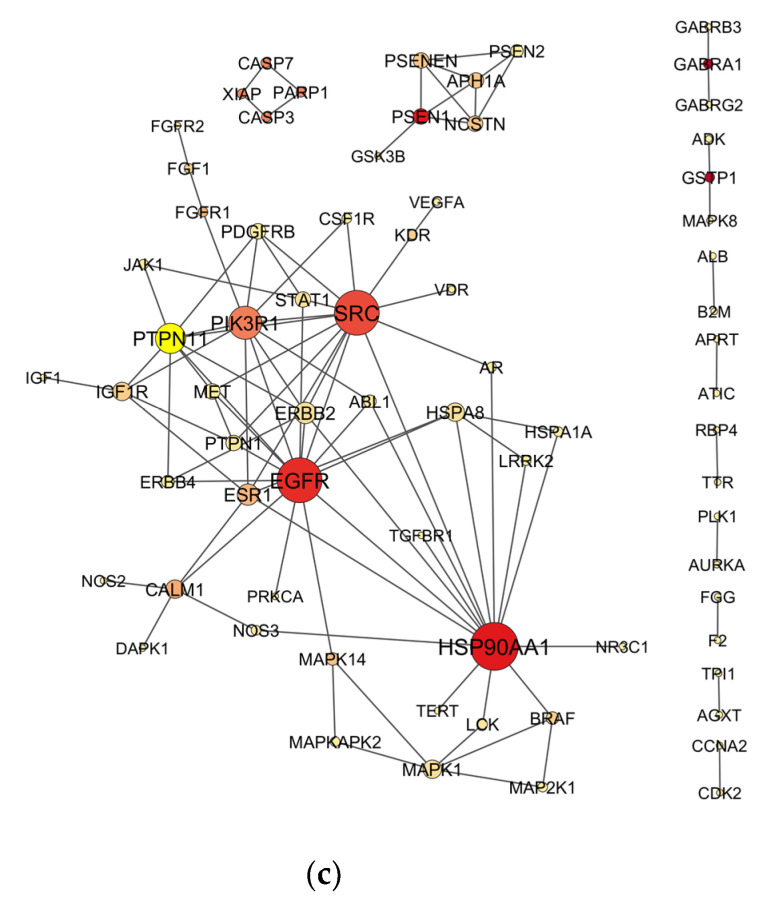
PPI Networks of organophosphate-induced developmental neurotoxicity: (**a**) diazinon-oxon, (**b**) chlorpyrifos oxon, (**c**) paraoxon. Each node represents a gene, and edges represent the interactions between them. The size of the node is directly related to the degree, and the color intensity represents the betweenness centrality of the nodes.

The analysis of PPI networks revealed 81 nodes and 113 edges for the DZO-NDT network, 37 nodes and 50 edges for the CPO-NDT network, and 71 nodes and 103 edges for the PO-NDT network (Figure 2). The top 10 hub nodes for each network were identified using the MCC algorithm in the cytoHubba plugin of Cytoscape. Topological measurements were obtained using the Network Analyzer tool. HSP90AA1, SRC, MET, and EGFR were hub nodes that were common among the three OPs. All hub nodes are listed in Table 2, along with their topological measurements. The measurements for the entire network are available in the supplementary materials (Tables S1–S3).

### 3.2. GO and Reactome Pathway Analysis

#### 3.2.1. GO Analysis

**Figure 3:**
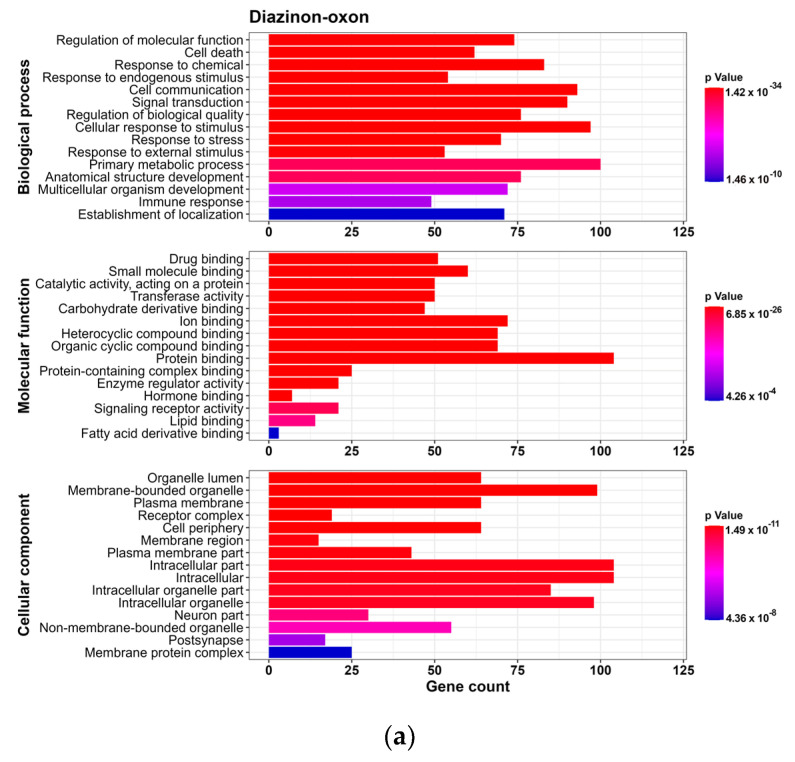

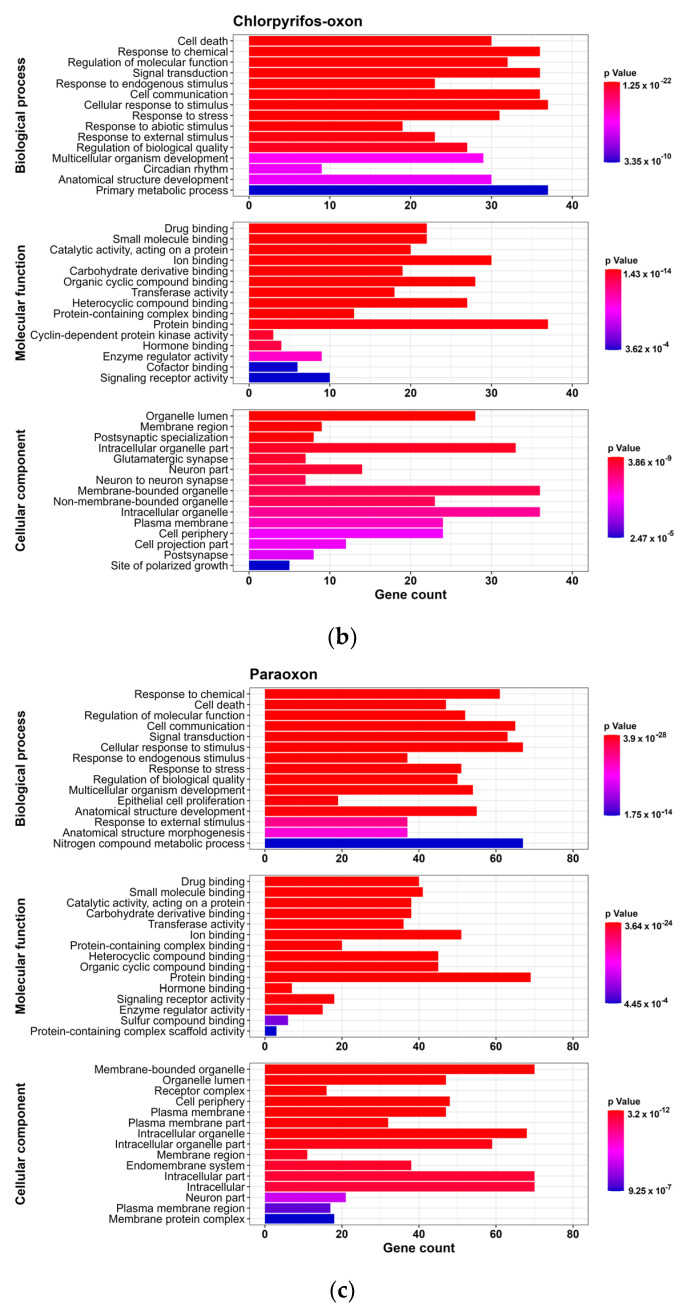
Results of Gene Ontology analyses for targets of Neurodevelopmental Toxicity induced by organophosphates (OPs) (**a**) diazinon-oxon, (**b**) chlorpyrifos oxon, and (**c**) paraoxon. The top 15 processes for Biological Process, Molecular Function, and Cellular Component for each OP were selected based on the *p*-value. The x-axis indicates the number of genes related to each process. The colors change from red to blue, indicating a decrease in the *p*-value.

To analyze the predicted NDT targets for each OP, we conducted a GO analysis for BP, CC and MF (*p*-value < 0.01). We found 169 GO terms for DZO, with 102 in BP, 20 in MF, and 47 in CC. For CPO, we identified 143 GO terms, with 89 in BP, 18 in MF, and 36 in CC. PO had 155 GO terms, with 98 in BP, 18 in MF, and 39 in CC. For each OP, we selected the Top 15 processes for each term based on their *p*-values (Figure 3).

We observed that the top 15 GO terms for BP, MF, and CC were similar for DZO, CPO, and PO. The results indicated that the BP terms were mainly related to cellular response to stress, which could be a result of chemical exposure, as well as cellular processes such as communication, signal transduction, and cell death, along with processes involved in the development of an anatomical structure or organism.

Regarding the MF terms, we found that most targets for the three OPs were associated with binding, which refers to the interaction between one molecule and specific sites of another molecule. The results of the CC terms suggested most targets were found within the intracellular milieu, organelles, plasma membrane, and neurons.

#### 3.2.2. Reactome Pathway Analysis

**Figure 4:**
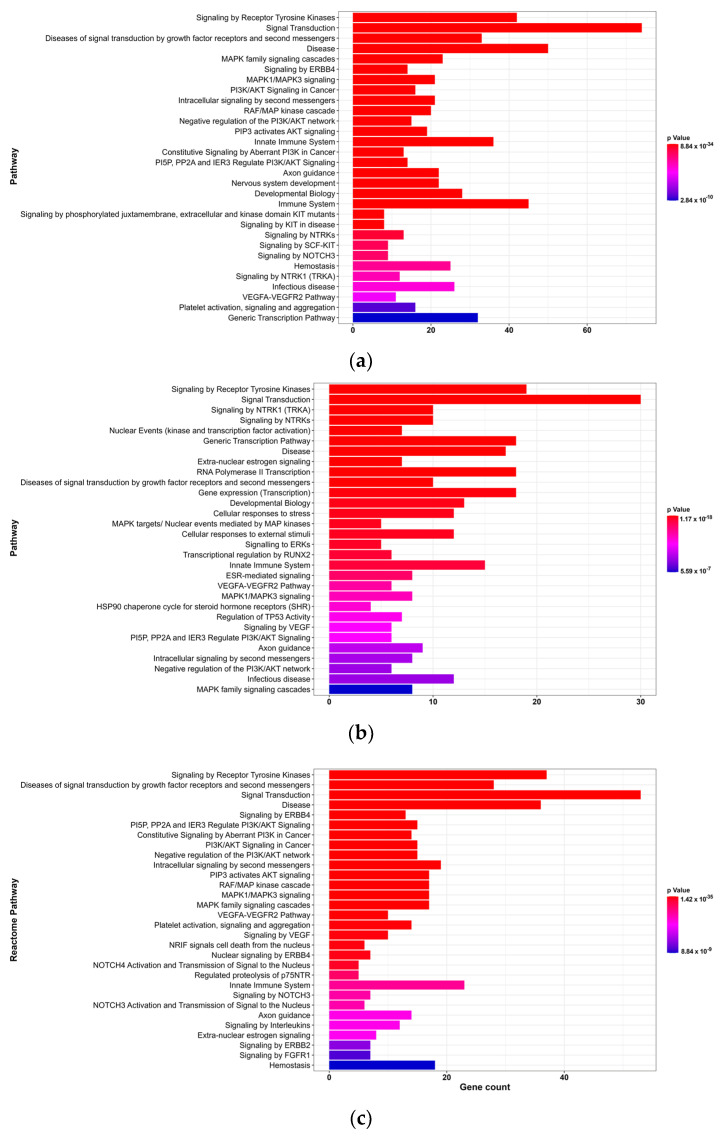
Results of reactome pathway analyses for targets of organophosphate-induced developmental neurotoxicity (**a**) diazinon-oxon, (**b**) chlorpyrifos oxon, and (**c**) paraoxon. The top 30 pathways based on *p*-value are shown. The x-axis represents the number of genes associated with each pathway. The colors transition from red to blue, indicating a decrease in *p*-value.

Based on Reactome pathway analysis, 419 pathways were identified for DZO, 254 for CPO, and 358 for PO (*p*-value < 0.01). Figure 4 shows the top 30 Reactome pathways for each OP based on the *p*-value.

Reactome pathway is a database that encompasses 2546 pathways organized into 28 superpathways (e.g., signal transduction, disease, immune system, and developmental biology) and their subdivisions [27]. According to the analyses, signal transduction was identified as the most important superpathway for DZO, CPO, and PO, which included pathways related to signaling by receptor tyrosine kinases, MAPK family signaling cascades, and intracellular signaling by second messengers. Additionally, all three OPs shared the axon guidance pathway, which is directly related to nervous system development, suggesting that OPs interfere with neurogenesis.

We also evaluated the Reactome pathways for DZO, CPO and PO that showed significant values (*p*-value < 0.01) and were classified under the Neuronal system superpathway to identify the key processes in the nervous system affected by OP exposure. Activation of NMDA receptors and postsynaptic events was the common pathway for all three OPs, indicating the activation of N-methyl-D-aspartate receptors (NMDAR), an ionotropic glutamate receptor.

### 3.3. Molecular Docking

**Figure 5:**
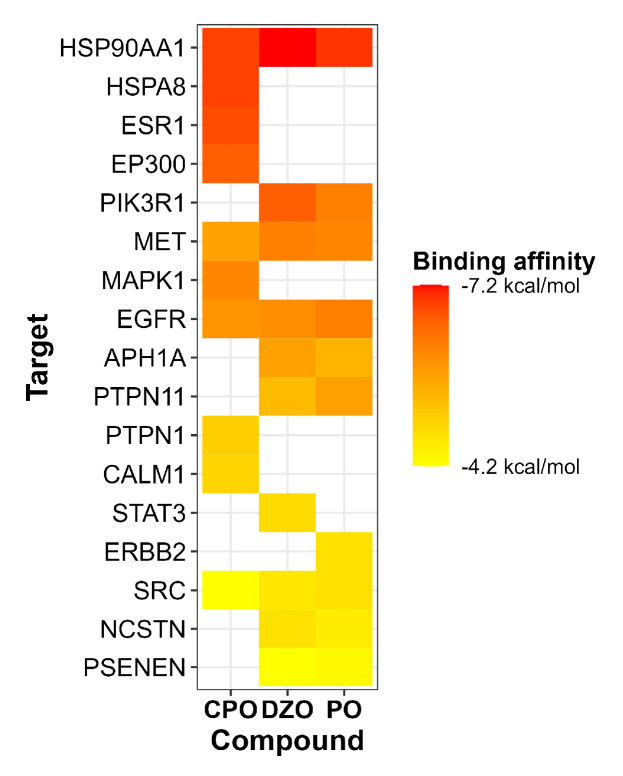
Heatmap of molecular docking of organophosphates (CPO, DZO, and PO) with their respective hub nodes, obtained from the PPI network analysis. The y-axis lists the hub nodes, and the x-axis lists the organophosphates. The color scale of the heatmap cells represents the predicted binding energy for each interaction. CPO: chlorpyrifos oxon, DZO: diazinon-oxon, PO: paraoxon.

We used molecular docking to predict the interactions between each OP and their respective hub nodes (presented in Table 2). A lower binding energy between the compound and the target indicates a higher affinity. The binding energies between the OPs and macromolecules ranged from −7.2 to −4.2 kcal/mol (Figure 5). Among the targets shared by the OPs, HSP90AA1 had the lowest binding energy with the OPs, followed by MET, EGFR, and SRC.

**Figure 6:**
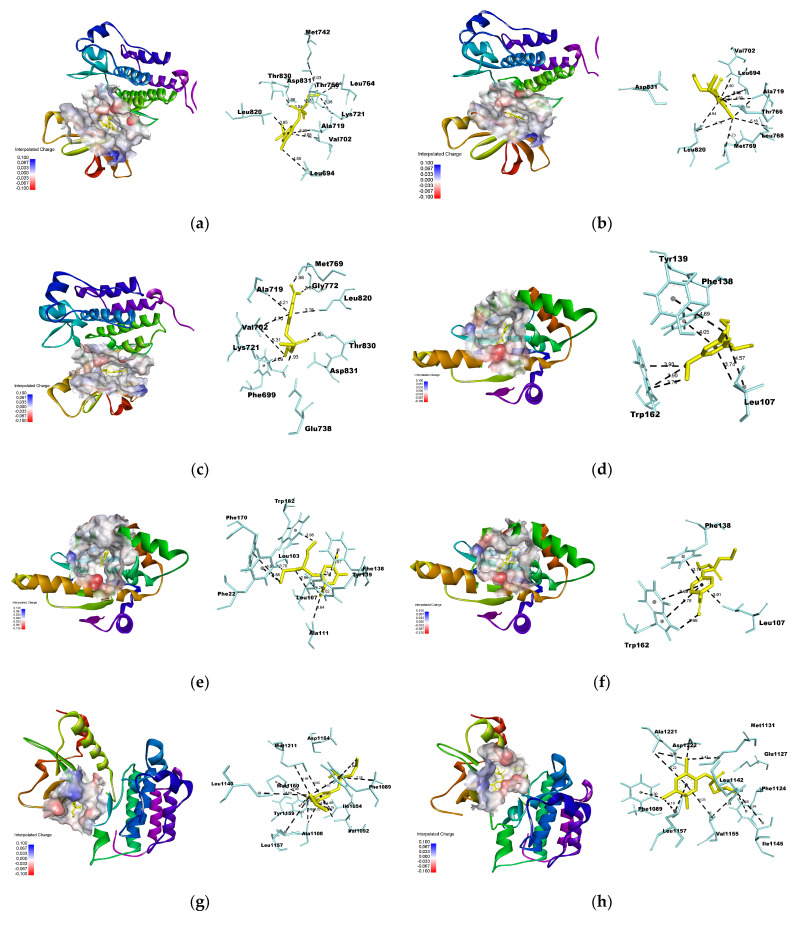

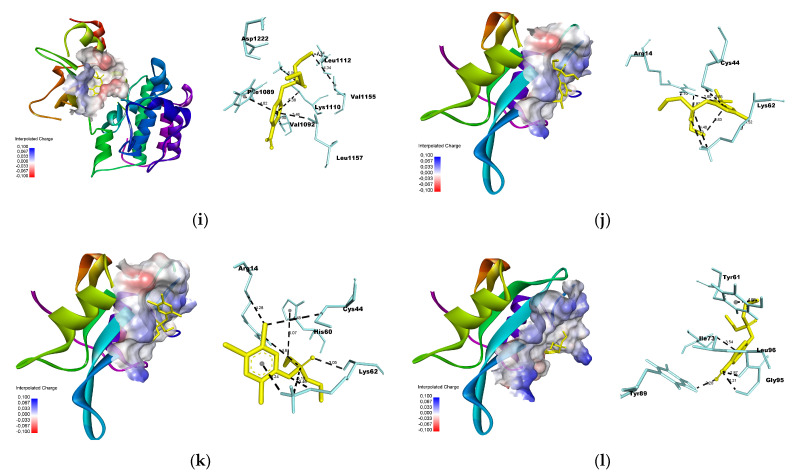
Interactions among the organophosphates diazinon-oxon (DZO), chlorpyrifos oxon (CPO), and paraoxon (PO) with the common hub nodes between them. (**a**–**c**) denote the interactions between EGFR and DZO, CPO, and PO, respectively. (**d**–**f**) represent the interactions between HSP90AA1 and DZO, CPO, and PO, respectively. (**g**–**i**) indicate the interactions between MET and DZO, CPO, and PO, respectively. (**j**–**l**) indicate the interactions between SRC and DZO, CPO, and PO, respectively.

The interactions between the OPs and the common hub nodes, EGFR, HSP90AA1, MET, and SRC, are shown in Figure 6. The OPs interacted with the ATP-binding site within the N-terminal domain of HSP90AA1. The ligands established π–sigma, π–alkyl and π–π interactions with specific amino acid residues. Similarly to PU3, an HSP90 inhibitor, the three OPs interacted with LEU^107^ and PHE^138^, and PO also formed hydrogen bonds with TRP^162^ [28]. The interactions between OPs and EGFR occurred in the ATP-binding cleft, positioned between the amino-terminal and carboxi-terminal lobes—an allosteric site. These interactions involved the formation of π-sigma, π-alkyl and hydrogen bonds with the amino acid residues. Similar to erlotinib, an EGFR inhibitor, the OPs interacted with MET^769^ and THR^766^ residues, as well as LYS^721^, recognized as one of the key residues for EGFR biological activity. Furthermore, all three OPs exhibited attractive forces with ASP^813^ residue of the catalytic loop [29,30].

OPs established multiple π-alkyl interactions with MET, in addition to forming π-sigma and π–π bonds. Interactions with residues at the ATP binding site were observed, including PHE^1089^ and VAL^1092^ in the N-lobe, and LEU^1157^ in the hinge region of the binding pocket. This region engages with non-competitive ATP inhibitors like Tivantinib [31,32]. The interactions between the OPs and the SRC SH2 domain occurred through hydrogen bonding, π-sigma, and π-alkyl interactions. DZO and CPO established hydrogen bonds with the LYS^62^ residue. Furthermore, DZO formed a hydrogen bond with ARG^14^ at the phosphotyrosine binding site. PO engaged in hydrogen bonding with the Leu^96^, Gly^95^, and Tyr^89^ residues within the specificity pocket [33].

Detailed results of the molecular docking analysis can be found in the Supplementary Materials (Tables S4 and S5 and Figure S1).

## 4. Discussion

The classic mechanism of neurotoxicity of organophosphates is the inhibition of the AChE activity, leading to the accumulation of the neurotransmitter acetylcholine in the synaptic cleft and consequently, overstimulation of cholinergic receptors. However, numerous studies have provided evidence that multiple mechanisms can contribute to the neurotoxicity of organophosphates, particularly during neurodevelopment [12].

Reactome pathway and Gene ontology enrichment analyzes were performed to identify potential mechanisms in the NTD of OP. The results indicate that these compounds impair signal transduction and axon guidance, disrupt cellular responses to stress, and activate NMDAR. In the OP-induced NTD PPI network, HSP90AA1, EGFR, MET, and SRC were identified as common hub nodes for DZO, CPO, and PO. For better understanding, the discussion will be divided into several topics according to the mechanism of toxicity.

### 4.1. Signal Transduction

Signal transduction is the process by which extracellular messengers bind to transmembrane receptors and provide information that triggers cellular responses, such as biochemical and biological transformations, as well as gene expression alterations [34]. According to Reactome pathway enrichment analyses, the major signal transduction pathways affected by organophosphates include the receptor Tyrosine Kinase (RTK) pathway, the mitogen-activated protein kinase (MAPK) pathway and signaling by second messengers. It is important to note that these pathways are interconnected, since RTK activation triggers phosphorylation and the recruitment of effector proteins, initiating signaling cascades involving MAPKs, PI3K/Akt, phospholipase C-PKC, and STAT [35].

In vitro studies have explored how OP exposure affects signaling pathways. For instance, exposure of cell cultures to CP and monocrotophos activated the MAPK ERK1/2, JNK, and p38 pathways, thereby inducing apoptosis. Activation of these pathways were associated with reactive oxygen species (ROS) generation and oxidative stress, which may contribute to mitochondrial damage and cell death [36,37,38]. It is plausible that these pathways may also be influenced by the interaction of OPs with RTKs.

Based on our literature review, no experimental data were found, indicating that OPs cause neurodevelopmental abnormalities via EGFR, MET, or non-receptor tyrosine kinase protein SRC. However, CP has previously been shown to increase migration and invasion of breast cancer cells through c-SRC pathway activation, amplifying downstream AKT and p-38 signaling [39]. Additionally, CP has promoted the growth of human colorectal adenocarcinoma H508 cells through increased EGFR/ERK1/2 signaling [40]. Although activation of the EGFR/ERK1/2 pathway generally stimulates cell growth, it can also promote neuronal cell death [41,42].

### 4.2. Axon Guidance

Axon guidance is the process by which neurons send their axons toward their correct targets for synaptic formation, and it is crucial during neural circuit development. Growing axons have a structure at their tips known as the growth cone, which contains receptors that respond to extracellular messengers that attract or repel the axon, guiding them to their destination [43]. The hub nodes EGFR, MET, and SRC play important roles in axon guidance.

The EGFR (or ErbB1) belongs to the ErbB family, which includes three other members: ErbB2, ErbB3, and ErbB4. Activation of EGFR initiates signaling pathways that are important for neuronal proliferation, differentiation, migration, as well as neural circuit development, including the Ras-Raf-MEK-ERK1/2, STAT3, and PI3K-Akt-mTOR pathways [44]. Additionally, activated EGFR interacts with SRC, forming a complex that enhances EGFR phosphorylation, consequently amplifying downstream signaling [45]. SRC also interacts with other signaling pathways involved in axon guidance, such as the Ephrin receptor (EphA) pathways [46], Sonic hedgehog (Shh) pathway [47], Netrin-1 pathway [48], and MET pathway [49]. Similar to EGFR, the activation of MET triggers downstream signaling pathways of PI3K/Akt, Ras-Raf-MEK-ERK, and STAT3, which are crucial for neurodevelopment and synaptogenesis [49].

Axonal growth is a fundamental process in the formation of neural circuits and the development of neuronal connectivity. Previous studies have reported impairment of axonal growth by OPs through noncholinergic mechanisms. For example, low concentrations of CP and CPO inhibited axonal outgrowth in cultures of rat sympathetic neurons [50]. Moreover, exposure to CPO during neurodevelopment disrupted axonal outgrowth in zebrafish [51]. These results showed that CP and CPO did not cause axon retraction or a decrease in the number of axons, but rather reduced growth rate compared to the untreated control group. Furthermore, no morphological changes in the axonal growth cone were observed. Experimental data suggest that OPs altered axonal growth signaling [50], similar to the findings in our study that OPs impair axon guidance.

### 4.3. Cellular Response to Stress

Exposure to environmental contaminants during prenatal development can trigger cellular response to stress that is closely related to heat shock and oxidative stress systems [52]. The *HSP90AA1* gene encodes the Heat shock protein (Hsps) HSP90, which acts as a chaperone to refold denatured proteins in the presence of stressors such as heat, oxidative stress, hypoxia, and exposure to cytotoxic agents. In the absence of stress, chaperones facilitate protein refolding, stabilization, translocation, and degradation, preventing protein aggregation. In neurodevelopment, Hsps regulate pathways related to cell growth and migration, such as the PI3K/Akt signaling pathway, and they are crucial mediators for axon guidance [53].

Cellular stress increases the expression of HSP90 and diverts its functions to cope with stress responses. This functional diversion or inhibition of HSP90 can alter the activity of various proteins and result in increased mutations, leading to neurodevelopmental disorders [54]. The increased expression of HSP90 has been observed in the liver, muscle, kidneys, and spleen of fish exposed to CP, indicating that OPs exposure can induce cellular response to stress [55,56]. Although these data were not obtained from nervous system cells, common carp exposed to CP showed increased expression of HSP90 and hypoactivity, which may indicate neurotoxicity [55].

There are several ways cells respond to stress, including activating of survival pathways or apoptosis [57]. There is still much to be clarified about the pathways by which OPs induce apoptosis, but they are known to occur during vulnerable windows of neurodevelopment and is likely not related to AChE inhibition [58]. However, it has been observed that the AKT pathway and oxidative stress are mechanisms by which OPs induce neural apoptosis, but independently of each other [59]. Therefore, the relationship between OPs and HSP90 may be a pathway to understanding how these processes occur.

### 4.4. Activation of NMDAR

The ionotropic glutamate receptors N-Methyl-D-Aspartate receptors (NMDAR) are calcium-permeable channels. The synaptic activity of NMDAR contributes to axonal and dendritic growth, as well as the maturation of glutamatergic synapses [60]. These processes are crucial for the development of cognitive functions, neuronal plasticity, memory, and learning [61]. Excessive activation of NMDAR can increase calcium influx, leading to cellular damage and neuronal death, likely mediated by the PKC/ERK pathway [62,63].

During exposure to high concentrations of OPs, ionotropic AMPA and NMDA glutamate receptors are activated due to increased excitatory signaling following AChE inhibition and excessive stimulation of muscarinic receptors, leading to seizures and neuronal death [64]. However, the mechanisms related to low concentrations of these chemicals have not been fully elucidated yet.

Studies indicate that glutamate-mediated excitotoxicity seems to be more related to the toxicity effects of CP than DZ. An NMDAR antagonist, MK-801, attenuated CP toxicity, but not DZ toxicity, in cortical neuronal and glial cultures [11]. Furthermore, in neonatal rats exposed to CP and DZ, both substances upregulated the expression of NMDAR subunits, but CP induced more significant changes than DZ [65].

In our study, we did not assess the direct activation of NMDAR by OPs, nor the genes encoding the subunits of this receptor. However, the activity of NMDAR can be regulated by SRC [66], EGFR [67], and MET [68], which have been identified as hub nodes in the PPI network of OPs. Furthermore, studies with chronic exposure to low doses of OPs is a more realistic way to assess environmental exposure, and further studies are needed to evaluate the relationship between this exposure and NMDAR activity, considering other biomarkers beyond the genes encoding the subunits of this receptor.

## 5. Conclusions

Using network toxicology and molecular docking analyses, this study identified the hub nodes HSP90AA1, EGFR, MET, and SRC as potential targets for OPs. Additionally, signal transduction, axon guidance, cellular response to stress, and the activation of NMDAR were found to be key pathways involved in OP-induced NDT. These findings are important for understanding the mechanisms of neurotoxicity of these substances at environmentally relevant concentrations that do not involve cholinergic pathways.

## Figures and Tables

**Table 1:** Grid box xyz coordinates for molecular docking between organophosphates DZO, CPO and PO and their respective hub nodes.

Molecule	Center Grid Box (xyz Coordinates)
HSP90AA1	6.63, 11.34, 24.85
HSPA8	17.27, −0.84, 2.30
ESR1	106.73, 15.02, 96.61
EP300	33.20, 9.43, −14.73
PIK3R1	−20.86, 10.90, 28.28
MET	61.53, 13.21, 117.38
MAPK1	−12.97, 13.19, 40.56
EGFR	22.01, 0.25, 52.79
APH1A	121.91, 88.97, 129.43
PTPN11	31.69, 2.17, 8.03
PTPN1	44.80, 14.03, 2.22
CALM1	3.70, 26.66, 105.09
STAT3	13.12, 55.60, 0.10
ERBB2	−21.17, 86.36, 138.72
SRC	19.73, 23.31, 21.53
NCSTN	113.91, 136.83, 145.71
PSENEN	184.59, 192.05, 152.23

**Table 2:** Topological measurements of hub nodes in the PPI networks of organophosphate-induced developmental neurotoxicity, identified using the MCC algorithm in the cytoHubba plugin of Cytoscape. k: degree; BC: Betweenness centrality; ASPL: Average shortest path length; CC: Closeness centrality.

Protein	Node	k	Clustering Coefficient	BC	ASPL	CC
Diazinon-oxon						
Proto-oncogene tyrosine kinase SRC	SRC	13	0.218	0.154	2.286	0.438
Epidermal growth factor receptor	EGFR	14	0.165	0.275	2.143	0.467
Phosphatidylinositol 3-kinase regulatory subunit alpha	PIK3R1	9	0.250	0.238	2.408	0.415
Tyrosine-protein phosphatase non-receptor type 11	PTPN11	7	0.4286	0.0164	2.7143	0.368
Heat shock protein HSP 90-alpha	HSP90AA1	11	0.109	0.154	2.429	0.412
Gamma-secretase subunit APH-1A	APH1A	4	0.8333	0.0667	1.5000	0.667
Nicastrin	NCSTN	4	0.833	0.067	1.500	0.667
Gamma-secretase subunit PEN-2	PSENEN	4	0.8333	0.0667	1.5000	0.667
Signal transducer and activator of transcription 3	STAT3	8	0.179	0.142	2.327	0.430
Hepatocyte growth factor receptor	MET	4	0.8333	0.0002	2.9184	0.343
Chlorpyrifos oxon						
Heat shock protein HSP 90-alpha	HSP90AA1	11	0.109	0.382	2.034	0.492
Epidermal growth factor receptor	EGFR	8	0.179	0.276	2.172	0.460
Proto-oncogene tyrosine kinase SRC	SRC					
Estrogen receptor	ESR1	7	0.143	0.269	2.103	0.475
Tyrosine-protein phosphatase non-receptor type 1	PTPN1	4	0.500	0.017	2.759	0.363
Hepatocyte growth factor receptor	MET	3	1.000	0.000	2.793	0.358
Mitogen-activated protein kinase1	MAPK1	5	0.100	0.095	3.172	0.315
Calmodulin 1	CALM1	5	0.000	0.158	2.414	0.414
Histone acetyltransferase p300	EP300	5	0.000	0.308	2.552	0.392
Heat shock cognate 71 kDa protein	HSPA8	3	0.667	0.008	2.379	0.420
Paraoxon						
Proto-oncogene tyrosine kinase SRC	SRC	14	0.220	0.264	1.875	0.533
Epidermal growth factor receptor	EGFR	14	0.198	0.312	1.800	0.556
Tyrosine-protein phosphatase non-receptor type 11	PTPN11	9	0.361	0.059	2.225	0.449
Phosphatidylinositol 3-kinase regulatory subunit alpha	PIK3R1	10	0.289	0.180	2.150	0.465
Steroid hormone receptor ERR2	ERRB2	6	0.667	0.026	2.100	0.476
Heat shock protein HSP 90-alpha	HSP90AA1	15	0.095	0.378	1.925	0.519
Gamma-secretase subunit APH-1A	APH1A	4	0.833	0.067	1.200	0.833
Nicastrin	NCSTN	4	0.833	0.067	1.200	0.833
Hepatocyte growth factor receptor	MET	4	0.833	0.001	2.450	0.408
Gamma-secretase subunit PEN-2	PSENEN	4	0.833	0.067	1.200	0.833

## Data Availability

The authors confirm that all data underlying the findings are fully available without restriction. All relevant data are within the paper.

## Supplementary Materials

The following supporting information can be downloaded at: https://www.mdpi.com/article/10.3390/toxics11080710/s1, Table S1: Topological measurements of nodes in the PPI network of diazinon oxon-induced developmental neurotoxicity, Table S2: Topological measurements of nodes in the PPI network of chlorpyrifos oxon-induced developmental neurotoxicity, Table S3: Topological measurements of nodes in the PPI network of paraoxon-induced developmental neurotoxicity, Table S4: Binding energies of molecular docking between organophosphates and their respective hub nodes, Table S5: Intermolecular interactions of complexes between HSP90AA1, EGFR, MET, and SRC and the organophosphates, Figure S1: Protein-ligand interactions between organophosphates and hub nodes generated using BIOVIA Discovery Studio Visualizer.
