# Supplementary material for: Network Toxicology and Molecular Docking to Investigate the Non-AChE Mechanisms of Organophosphate-Induced Neurodevelopmental Toxicity
Source: Toxics. 2023 Aug 17;11(8):710. doi: 10.3390/toxics11080710 (PMC10458981; doi:10.3390/toxics11080710)
Supplement: Supplementary file 1 [file toxics-11-00710-s001.zip › toxics-2521608-supplementary.pdf]

toxics MDPI

Table S1. - Topological measurements of nodes in the PPI network of diazinon oxon-induced developmental neurotoxicity.

Protein	Node	k	Clustering coefficient	BC	ASPL	CC
Epidermal growth factor receptor	EGFR	14	0.1648	0.2748	2.1429	0.4667
Proto-oncogene tyrosine kinase SRC	SRC	13	0.2179	0.1544	2.2857	0.4375
Heat shock protein HSP 90-alpha	HSP90AA1	11	0.1091	0.1543	2.4286	0.4118
Histone acetyltransferase p300	EP300	9	0.0278	0.3026	2.4898	0.4016
Phosphatidylinositol 3-kinase regulatory subunit alpha	PIK3R1	9	0.2500	0.2378	2.4082	0.4153
**Signal transducer and activator of transcription 3**	STAT3	8	0.1786	0.1418	2.3265	0.4298
Estrogen receptor	ESR1	7	0.1905	0.2018	2.2449	0.4455
Calmodulin-1	CALM1	7	0.0000	0.1683	2.6531	0.3769
Tyrosine-protein phosphatase non-receptor type 11	PTPN11	7	0.4286	0.0164	2.7143	0.3684
Insulin-like growth factor 1 receptor	IGF1R	6	0.1333	0.0556	2.5918	0.3858
Mitogen-activated protein kinase1	MAPK1	6	0.2000	0.0520	3.0612	0.3267
Signal transducer and activator of transcription 1-alpha/beta	STAT1	5	0.5000	0.0375	2.4694	0.4050
Presenilin	PSEN1	4	0.5000	0.5333	1.3333	0.7500
Nicastrin	NCSTN	4	0.8333	0.0667	1.5000	0.6667
Gamma-secretase subunit APH-1A	APH1A	4	0.8333	0.0667	1.5000	0.6667
Gamma-secretase subunit PEN-2	PSENEN	4	0.8333	0.0667	1.5000	0.6667
Tyrosine-protein phosphatase non-receptor type 1	PTPN1	4	0.5000	0.0036	2.8980	0.3451
Platelet-derived growth factor receptor beta	PDGFRB	4	0.6667	0.0030	2.8163	0.3551
Hepatocyte growth factor receptor	MET	4	0.8333	0.0002	2.9184	0.3427
Cell division control protein 42 homolog	CDC42	3	0.0000	0.1556	3.2245	0.3101
**Poly [ADP-ribose] polymerase 1**	PARP1	3	0.0000	0.1178	3.3469	0.2988
Mitogen-activated protein kinase14	MAPK14	3	0.3333	0.0312	2.9592	0.3379
Androgen receptor	AR	3	0.3333	0.0230	2.6939	0.3712
Serine/threonine-protein kinase B-raf	BRAF	3	0.3333	0.0185	3.2449	0.3082
Tyrosine-protein kinase ABL1	ABL1	3	0.6667	0.0079	2.7347	0.3657
Tyrosine-protein kinase Lck	LCK	3	0.3333	0.0037	3.0000	0.3333
Heat Shock cognate 71 kDa protein	HSPA8	3	0.6667	0.0007	2.6939	0.3712
Presenilin-2	PSEN2	3	1.0000	0.0000	2.0000	0.5000
**C-C motif chemokine 5**	CCL5	2	0.0000	1.0000	1.0000	1.0000
**Gamma-aminobutyric acid receptor subunit alpha-1**	GABRA1	2	0.0000	1.0000	1.0000	1.0000
**Glycogen synthase kinase-3 beta**	GSK3B	2	0.0000	0.3333	1.8333	0.5455
Mitogen-activated protein kinase8	MAPK8	2	0.0000	0.0799	4.1224	0.2426
**Glutathione S-transferase P**	GSTP1	2	0.0000	0.0408	5.0612	0.1976
Cyclin-dependent kinase 1	CDK1	2	0.0000	0.0300	3.0408	0.3289
**Caspase-7**	CASP7	2	0.0000	0.0200	4.2857	0.2333
**Caspase-3**	CASP3	2	0.0000	0.0200	4.2857	0.2333
Cyclin-dependent kinase 2	CDK2	2	0.0000	0.0134	3.3878	0.2952
**Nitric oxide synthase, endotelial**	NOS3	2	0.0000	0.0079	3.2041	0.3121
**Cyclin-A2**	CCNA2	2	0.0000	0.0043	3.6122	0.2768

Glucocorticoid receptor	NR3C1	2	0.0000	0.0005	3.0816	0.3245
**E3 ubiquitin-protein ligase XIAP**	XIAP	2	0.0000	0.0004	5.2245	0.1914
**Dual specificity mitogen-activated protein kinase kinase 1**	MAP2K1	2	1.0000	0.0000	3.8776	0.2579
Receptor tyrosine-protein kinase erbB-4	ERBB4	2	1.0000	0.0000	3.0408	0.3289
MAP kinase-activated protein kinase 2	MAPKAPK2	2	1.0000	0.0000	3.5918	0.2784
**Annexin A5**	ANXA5	1	0.0000	0.0000	1.0000	1.0000
**Arachidonate 5-lipoxygenase-activating protein**	ALOX5AP	1	0.0000	0.0000	1.0000	1.0000
**Bcl-2-like protein 1**	BCL2L1	1	0.0000	0.0000	1.0000	1.0000
**Retinol-binding protein 4**	RBP4	1	0.0000	0.0000	1.0000	1.0000
**Polyunsaturated fatty acid 5-lipoxygenase**	ALOX5	1	0.0000	0.0000	1.0000	1.0000
Adenosine kinase	ADK	1	0.0000	0.0000	6.0408	0.1655
**Aurora kinase A**	AURKA	1	0.0000	0.0000	1.0000	1.0000
**TGF-beta receptor type-1**	TGFBR1	1	0.0000	0.0000	3.4082	0.2934
**Serine/threonine-protein kinase PLK1**	PLK1	1	0.0000	0.0000	1.0000	1.0000
**Bifunctional purine biosynthesis protein ATIC**	ATIC	1	0.0000	0.0000	1.0000	1.0000
**Beta-2-microglobulin**	B2M	1	0.0000	0.0000	1.0000	1.0000
**Histone acetyltransferase KAT2B**	KAT2B	1	0.0000	0.0000	3.4694	0.2882
Peroxisome proliferator-activated receptor gamma	PPARG	1	0.0000	0.0000	3.4694	0.2882
Cyclin-dependent kinase 5 activator 1	CDK5R1	1	0.0000	0.0000	1.0000	1.0000
**Triosephosphate isomerase**	TPI1	1	0.0000	0.0000	1.0000	1.0000
**Apoptotic protease-activating factor 1**	APAF1	1	0.0000	0.0000	1.0000	1.0000
**Transthyretin**	TTR	1	0.0000	0.0000	1.0000	1.0000
**Fibroblast growth factor receptor 1**	FGFR1	1	0.0000	0.0000	3.3878	0.2952
**Gamma-aminobutyric acid receptor subunit gamma-2**	GABRG2	1	0.0000	0.0000	1.5000	0.6667
**Gamma-aminobutyric acid receptor subunit beta-3**	GABRB3	1	0.0000	0.0000	1.5000	0.6667
**Adenine phosphoribosyltransferase**	APRT	1	0.0000	0.0000	1.0000	1.0000
**Alanine--glyoxylate aminotransferase**	AGXT	1	0.0000	0.0000	1.0000	1.0000
Cyclin-dependent kinase 5	CDK5	1	0.0000	0.0000	1.0000	1.0000
**Death-associated protein kinase 1**	DAPK1	1	0.0000	0.0000	3.6327	0.2753
**Insulin-like growth factor I**	IGF1	1	0.0000	0.0000	3.5714	0.2800
**Peroxisome proliferator-activated receptor alpha**	PPARA	1	0.0000	0.0000	3.4694	0.2882
**Serine/threonine-protein kinase PAK 6**	PAK6	1	0.0000	0.0000	4.2041	0.2379
**Protein S100-A9**	S100A9	1	0.0000	0.0000	1.0000	1.0000
**Vascular endothelial growth factor receptor 2**	KDR	1	0.0000	0.0000	3.2653	0.3062
**C-C chemokine receptor type 1**	CCR1	1	0.0000	0.0000	1.5000	0.6667
Nitric oxide synthase, inducible	NOS2	1	0.0000	0.0000	3.6327	0.2753
**RAC-beta serine/threonine-protein kinase**	AKT2	1	0.0000	0.0000	2.6667	0.3750
Albumin	ALB	1	0.0000	0.0000	1.0000	1.0000
**Voltage-dependent L-type calcium channel subunit alpha-1C**	CACNA1C	1	0.0000	0.0000	3.6327	0.2753
**C-C chemokine receptor type 5**	CCR5	1	0.0000	0.0000	1.5000	0.6667
**Vitamin D3 receptor**	VDR	1	0.0000	0.0000	3.2653	0.3062
Nitric oxide synthase, brain	NOS1	1	0.0000	0.0000	3.6327	0.2753

Table S2. - Topological measurements of nodes in the PPI network of chlorpyrifos oxon-induced developmental neurotoxicity.

Protein	Node	k	Clustering coefficient	BC	ASPL	CC
Heat shock protein HSP 90-alpha	HSP90AA1	11	0.109	0.382	2.034	0.492
Epidermal growth factor receptor	EGFR	8	0.179	0.276	2.172	0.460
Estrogen receptor	ESR1	7	0.143	0.269	2.103	0.475
Proto-oncogene tyrosine kinase SRC	SRC	6	0.400	0.071	2.276	0.439
Histone acetyltransferase p300	EP300	5	0.000	0.308	2.552	0.392
Calmodulin-1	CALM1	5	0.000	0.158	2.414	0.414
Mitogen-activated protein kinase1	MAPK1	5	0.100	0.095	3.172	0.315
Tyrosine-protein phosphatase non-receptor type 1	PTPN1	4	0.500	0.017	2.759	0.363
**Poly [ADP-ribose] polymerase 1**	PARP1	3	0.000	0.135	3.379	0.296
Mitogen-activated protein kinase14	MAPK14	3	0.333	0.090	2.862	0.349
Androgen receptor	AR	3	0.333	0.088	2.448	0.408
Heat Shock cognate 71 kDa protein	HSPA8	3	0.667	0.008	2.379	0.420
Hepatocyte growth factor receptor	MET	3	1.000	0.000	2.793	0.358
Adenosine receptor A2a	ADORA2A	2	0.000	1.000	1.000	1.000
Cyclin-dependent kinase 1	CDK1	2	0.000	0.057	2.828	0.354
Tyrosine-protein kinase Lck	LCK	2	0.000	0.044	2.759	0.363
Serine/threonine-protein kinase B-raf	BRAF	2	0.000	0.044	2.759	0.363
Cyclin-dependent kinase 2	CDK2	2	0.000	0.032	3.276	0.305
**Cyclin-A2**	CCNA2	2	0.000	0.025	3.310	0.302
**Nitric oxide synthase, endotelial**	NOS3	2	0.000	0.014	2.793	0.358
Insulin-like growth factor 1 receptor	IGF1R	2	0.000	0.007	2.828	0.354
MAP kinase-activated protein kinase 2	MAPKAPK2	2	1.000	0.000	3.690	0.271
Aryl hydrocarbon receptor	AHR	2	1.000	0.000	2.621	0.382
Cyclin-dependent kinase 5	CDK5	1	0.000	0.000	1.000	1.000
D(2) dopamine receptor	DRD2	1	0.000	0.000	1.500	0.667
**TGF-beta receptor type-1**	TGFBR1	1	0.000	0.000	3.000	0.333
**Glutathione S-transferase P**	GSTP1	1	0.000	0.000	1.000	1.000
Dual specific protein phosphatase 6	DUSP6	1	0.000	0.000	4.138	0.242
Cannabinoid receptor 1	CNR1	1	0.000	0.000	1.500	0.667
Nitric oxide synthase, inducible	NOS2	1	0.000	0.000	3.379	0.296
Glucocorticoid receptor	NR3C1	1	0.000	0.000	3.000	0.333
Peroxisome proliferator-activated receptor gamma	PPARG	1	0.000	0.000	3.517	0.284
Cyclin-dependent kinase 5 activator 1	CDK5R1	1	0.000	0.000	1.000	1.000
Nitric oxide synthase, brain	NOS1	1	0.000	0.000	3.379	0.296
Mitogen-activated protein kinase8	MAPK8	1	0.000	0.000	1.000	1.000
**Caspase-7**	CASP7	1	0.000	0.000	4.345	0.230
**Caspase-3**	CASP3	1	0.000	0.000	4.345	0.230

Table S3. - Topological measurements of nodes in the PPI network of paraoxon-induced developmental neurotoxicity.

Protein	Node	k	Clustering coefficient	BC	ASPL	CC
Heat shock protein HSP 90-alpha	HSP90AA1	15	0.095	0.378	1.925	0.519
Epidermal growth factor receptor	EGFR	14	0.198	0.312	1.800	0.556
Proto-oncogene tyrosine kinase SRC	SRC	14	0.220	0.264	1.875	0.533

Phosphatidylinositol 3-kinase regulatory subunit alpha	PIK3R1	10	0.289	0.180	2.150	0.465
Tyrosine-protein phosphatase non-receptor type 11	PTPN11	9	0.361	0.059	2.225	0.449
Estrogen receptor	ESR1	6	0.267	0.091	2.150	0.465
Receptor tyrosine-protein kinase erbB-2	ERBB2	6	0.667	0.026	2.100	0.476
Calmodulin-1	CALM1	5	0.000	0.108	2.525	0.396
Insulin-like growth factor 1 receptor	IGF1R	5	0.200	0.054	2.550	0.392
Mitogen-activated protein kinase1	MAPK1	5	0.200	0.021	3.150	0.317
Heat Shock cognate 71 kDa protein	HSPA8	5	0.400	0.021	2.350	0.426
Presenilin	PSEN1	4	0.500	0.400	1.200	0.833
Gamma-secretase subunit APH-1A	APH1A	4	0.833	0.067	1.200	0.833
Nicastrin	NCSTN	4	0.833	0.067	1.200	0.833
Gamma-secretase subunit PEN-2	PSENEN	4	0.833	0.067	1.200	0.833
Signal transducer and activator of transcription 1-alpha/beta	STAT1	4	0.333	0.019	2.500	0.400
Tyrosine-protein phosphatase non-receptor type 1	PTPN1	4	0.500	0.009	2.450	0.408
Platelet-derived growth factor receptor beta	PDGFRB	4	0.667	0.003	2.575	0.388
Hepatocyte growth factor receptor	MET	4	0.833	0.001	2.450	0.408
Mitogen-activated protein kinase14	MAPK14	3	0.333	0.069	2.575	0.388
Serine/threonine-protein kinase B-raf	BRAF	3	0.333	0.052	2.725	0.367
Tyrosine-protein kinase ABL1	ABL1	3	0.667	0.009	2.275	0.440
Presenilin-2	PSEN2	3	1.000	0.000	1.600	0.625
Receptor tyrosine-protein kinase erbB-4	ERBB4	3	1.000	0.000	2.625	0.381
**Glutathione S-transferase P**	GSTP1	2	0.000	1.000	1.000	1.000
**Gamma-aminobutyric acid receptor subunit alpha-1**	GABRA1	2	0.000	1.000	1.000	1.000
**Caspase-3**	CASP3	2	0.000	0.167	1.333	0.750
**Poly [ADP-ribose] polymerase 1**	PARP1	2	0.000	0.167	1.333	0.750
**E3 ubiquitin-protein ligase XIAP**	XIAP	2	0.000	0.167	1.333	0.750
**Caspase-7**	CASP7	2	0.000	0.167	1.333	0.750
**Fibroblast growth factor receptor 1**	FGFR1	2	0.000	0.097	3.025	0.331
**Fibroblast growth factor 1**	FGF1	2	0.000	0.050	3.950	0.253
**Vascular endothelial growth factor receptor 2**	KDR	2	0.000	0.050	2.800	0.357
Tyrosine-protein kinase Lck	LCK	2	0.000	0.013	2.750	0.364
**Nitric oxide synthase, endotelial**	NOS3	2	0.000	0.011	2.750	0.364
**Tyrosine-protein kinase JAK1**	JAK1	2	0.000	0.001	3.150	0.317
Androgen receptor	AR	2	1.000	0.000	2.525	0.396
**Dual specificity mitogen-activated protein kinase kinase 1**	MAP2K1	2	1.000	0.000	3.600	0.278
**Macrophage colony-stimulating factor 1 receptor**	CSF1R	2	1.000	0.000	2.675	0.374
**Leucine-rich repeat serine/threonine-protein kinase 2**	LRRK2	2	1.000	0.000	2.875	0.348
**Heat shock 70 kDa protein 1A**	HSPA1A	2	1.000	0.000	2.875	0.348
MAP kinase-activated protein kinase 2	MAPKAPK2	2	1.000	0.000	3.450	0.290
Adenosine kinase	ADK	1	0.000	0.000	1.500	0.667
**Alanine--glyoxylate aminotransferase**	AGXT	1	0.000	0.000	1.000	1.000
**Triosephosphate isomerase**	TPI1	1	0.000	0.000	1.000	1.000

Albumin	ALB	1	0.000	0.000	1.000	1.000
**Beta-2-microglobulin**	B2M	1	0.000	0.000	1.000	1.000
**Adenine phosphoribosyltransferase**	APRT	1	0.000	0.000	1.000	1.000
**Bifunctional purine biosynthesis protein ATIC**	ATIC	1	0.000	0.000	1.000	1.000
**Aurora kinase A**	AURKA	1	0.000	0.000	1.000	1.000
**Serine/threonine-protein kinase PLK1**	PLK1	1	0.000	0.000	1.000	1.000
Nitric oxide synthase, inducible	NOS2	1	0.000	0.000	3.500	0.286
**Death-associated protein kinase 1**	DAPK1	1	0.000	0.000	3.500	0.286
**Cyclin-A2**	CCNA2	1	0.000	0.000	1.000	1.000
Cyclin-dependent kinase 2	CDK2	1	0.000	0.000	1.000	1.000
**Protein kinase C alpha type**	PRKCA	1	0.000	0.000	2.775	0.360
**Prothrombin**	F2	1	0.000	0.000	1.000	1.000
**Fibrinogen gamma chain**	FGG	1	0.000	0.000	1.000	1.000
**Fibroblast growth factor receptor 2**	FGFR2	1	0.000	0.000	4.925	0.203
**Gamma-aminobutyric acid receptor subunit beta-3**	GABRB3	1	0.000	0.000	1.500	0.667
**Gamma-aminobutyric acid receptor subunit gamma-2**	GABRG2	1	0.000	0.000	1.500	0.667
**Glycogen synthase kinase-3 beta**	GSK3B	1	0.000	0.000	2.000	0.500
Mitogen-activated protein kinase8	MAPK8	1	0.000	0.000	1.500	0.667
Glucocorticoid receptor	NR3C1	1	0.000	0.000	2.900	0.345
**Telomerase reverse transcriptase**	TERT	1	0.000	0.000	2.900	0.345
TGF-beta receptor type-1	TGFBR1	1	0.000	0.000	2.900	0.345
**Insulin-like growth factor I**	IGF1	1	0.000	0.000	3.525	0.284
**Vascular endothelial growth factor A**	VEGFA	1	0.000	0.000	3.775	0.265
**Retinol-binding protein 4**	RBP4	1	0.000	0.000	1.000	1.000
**Transthyretin**	TTR	1	0.000	0.000	1.000	1.000
**Vitamin D3 receptor**	VDR	1	0.000	0.000	2.850	0.351

Table S4. - Binding energies of molecular docking between organophosphates and their respective hub nodes.

Target	PDB entry	Ligand	Docking energy (kJ/mol)
HSP90AA1	1UY9	PU6*	-9.0
		Diazinon oxon	-7.2
		Chlorpyrifos oxon	-6.9
		Paraoxon	-7.0
HSPA8	3FZK	3BK*	-8.5
		Chlorpyrifos oxon	-6.9
ESR1	1A52	EST*	-10.4
		Chlorpyrifos oxon	-6.8
EP300	6V8N	QS1*	-12.2
		Chlorpyrifos oxon	-6.6
PIK3R1	3ZIM	KKR*	-9.6
		Diazinon oxon	-6.6
		Paraoxon	-6.2
MET	2RFN	AM7*	-11.1
		Diazinon oxon	-6.2
		Chlorpyrifos oxon	-5.7
		Paraoxon	-6.1

MAPK1	1PME	SB2*	-8.4
		Chlorpyrifos oxon	-6.1
EGFR	1M17	AQ4*	-7.2
		Diazinon oxon	-6.0
		Chlorpyrifos oxon	-5.9
		Paraoxon	-6.2
APH1A	5A63	PC1*	-4.8
		Diazinon oxon	-5.7
		Paraoxon	-5.4
PTPN11	3MOW	B2B*	-6.0
		Diazinon oxon	-5.3
		Paraoxon	-5.7
CALM1	1CTR	TFP*	-7.5
		Chlorpyrifos oxon	-4.9
STAT3	6NUQ	KQV*	-6.7
		Diazinon oxon	-4.8
ERBB2	1N8Z	NAG*	-4.9
		Paraoxon	-4.7
SRC	1O46	903*	-7.7
		Diazinon oxon	-4.6
		Chlorpyrifos oxon	-4.2
		Paraoxon	-4.7
NCSTN	5A63	NAG*	-4.7
		Diazinon oxon	-4.7
		Paraoxon	-4.5
PSENEN	6IDF	NAG*	-4.4
		Diazinon oxon	-4.2
		Paraoxon	-4.3
PTPN1	1C84	761*	-8.1
		Chlorpyrifos oxon	-5.0

*Original ligand

Table S5. - Intermolecular interactions of complexes between HSP90AA1, EGFR, MET, and SRC and the organophosphates.

Complex	Hydrogen bond (HB) interaction	Distance (Å) for HB interaction	Carbon-hydrogen interaction	Pi-sigma interaction	Pi-Alkyl interaction	Pi-Pi interaction
HSP90AA1 – DZO				Trp162, Leu107	Tyr139	Phe138
HSP90AA1 – CPO			Leu103	Leu107	Phe22, Phe170, Ala111	Phe138
HSP90AA1 – PO	Trp162	2.66, 5.09,5.78		Leu107		Phe138
EGFR-DZO	Thr830<br>Thr766	1.99<br>2.26		Leu820	Met742, Lys721, Leu764, Leu 694, Val702, Ala719	

EGFR-CPO	Thr766<br>Met769	2.04<br>4.63		Val702	Ala719,<br>Leu768,<br>Leu820,<br>Leu694	
EGFR-PO	Lys721<br>Met769<br>Thr830	1.93<br>1.96<br>2.4	Gly772	Leu820	Phe699, Val,<br>702, Ala719	
MET-DZO				Val1092,<br>Met1211,<br>Phe1089	Tyr1159,<br>Met1160,<br>Ala1108,<br>Leu1140,<br>Ile1084,<br>Leu1157	
MET-CPO					Ile1145,<br>Leu1142,<br>Phe1124,<br>Met1131,<br>Ala1221,<br>Val1155,<br>Phe1089,<br>Leu1157	
MET-PO				Leu1157	Val1092,<br>Val1155,<br>Leu1112	Phe1089
SRC-DZO	Lys62<br>Arg14	2.73, 4.52<br>2.10, 2.82				
SRC-CPO	Lys62	3.5			His60, Cys44	
SRC- PO	Leu96<br>Gly95<br>Tyr89	2.26<br>2.21<br>2.87		Ile73	Tyr61	

Figure S1. - Protein-ligand interactions between organophosphates and hub nodes generated using BIOVIA Discovery Studio Visualizer.

HSP90AA1

PU6*

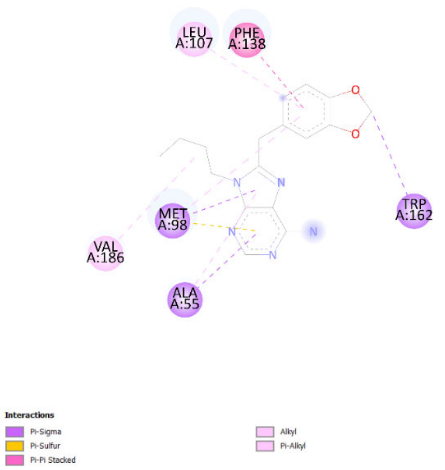

Diazinon oxon

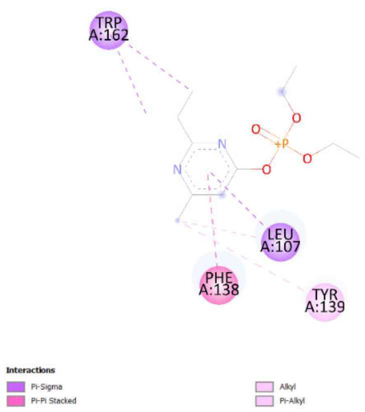

Chlorpyrifos oxon

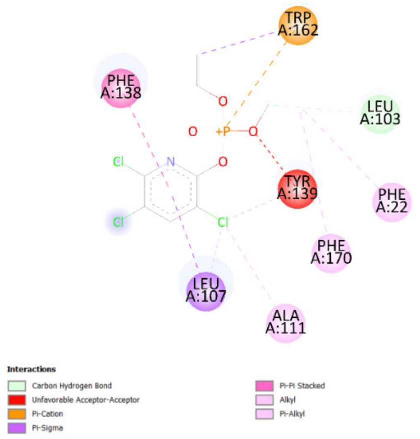

Paraoxon

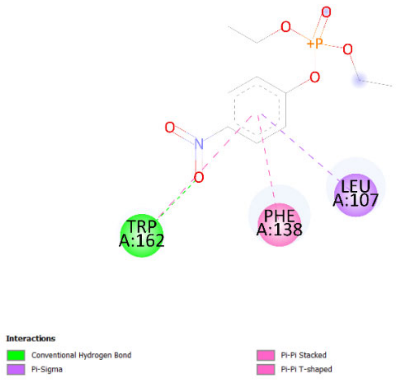

HSPA8

2BK*

Chlorpyrifos oxon

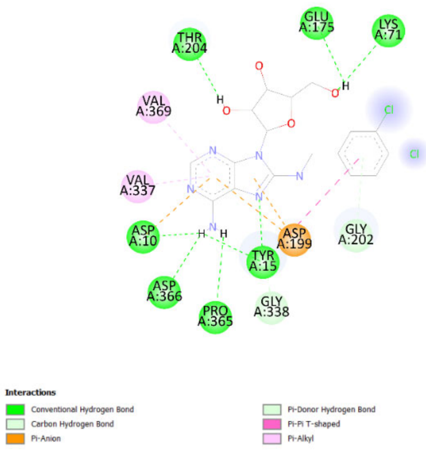

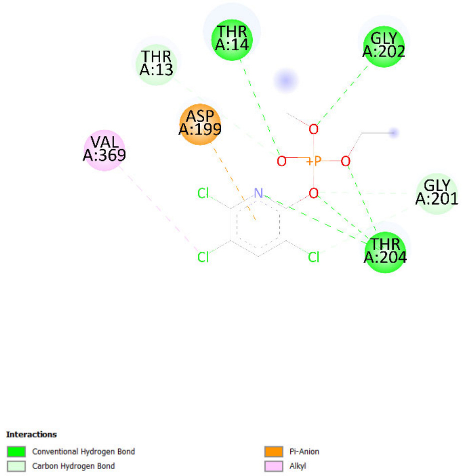

ESR1

EST*

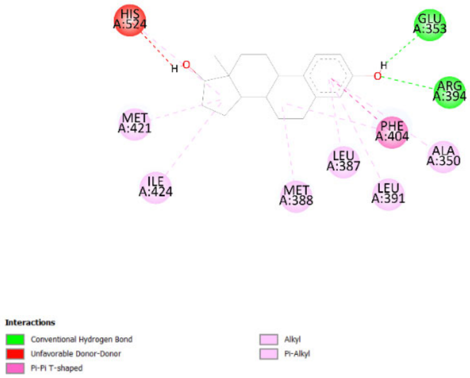

Chlorpyrifos oxon

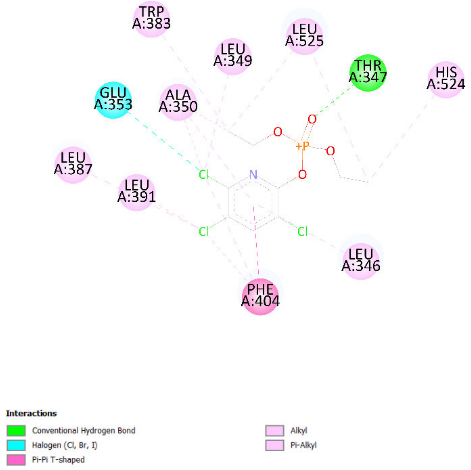

EP300

QS1*

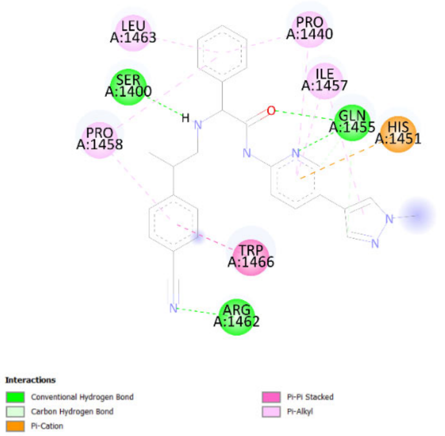

Chlorpyrifos oxon

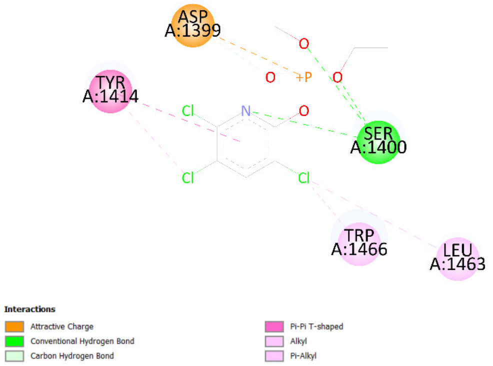

PIK3R1

KKR*

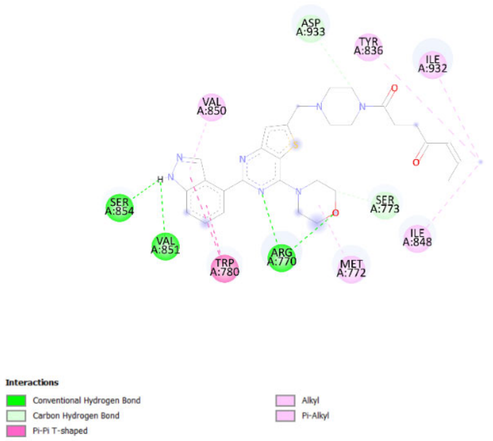

Diazinon oxon

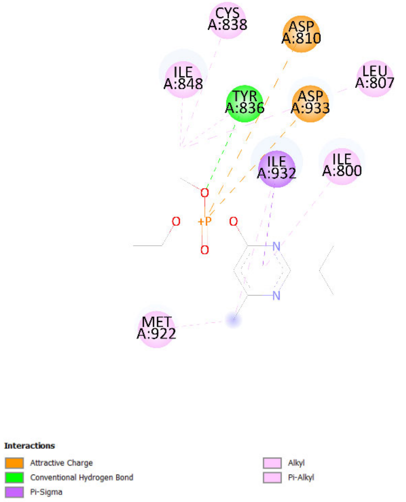

Paraoxon

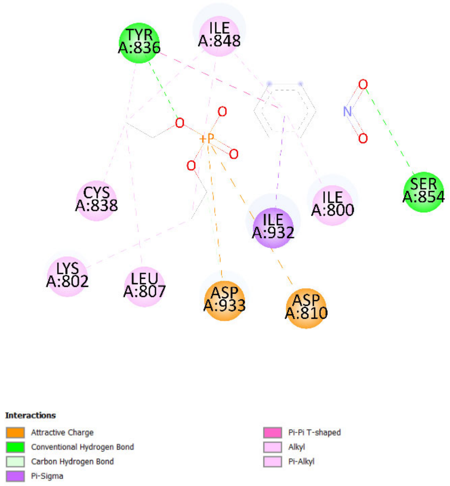

MET

AM7*

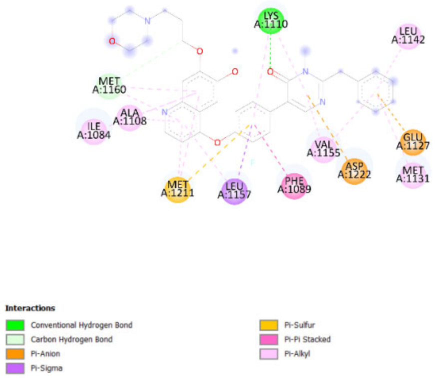

Diazinon oxon

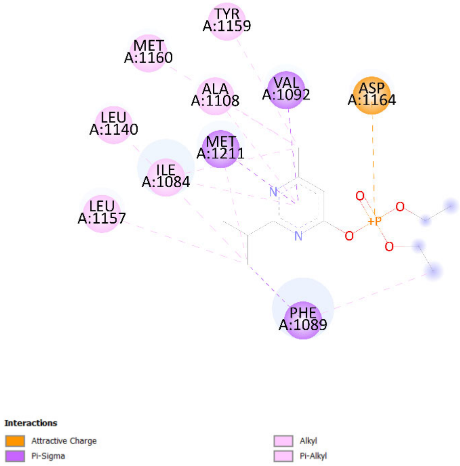

Chlorpyrifos oxon

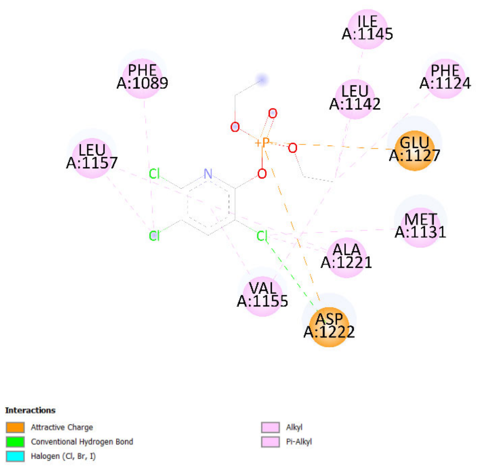

Paraoxon

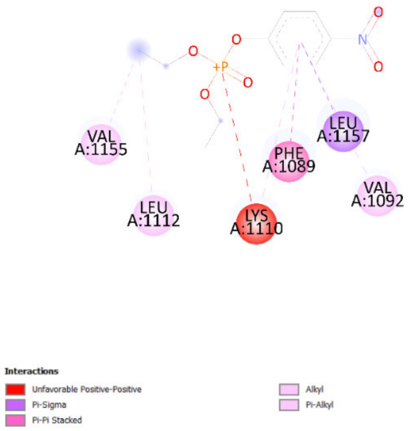

MAPK1

SB2*

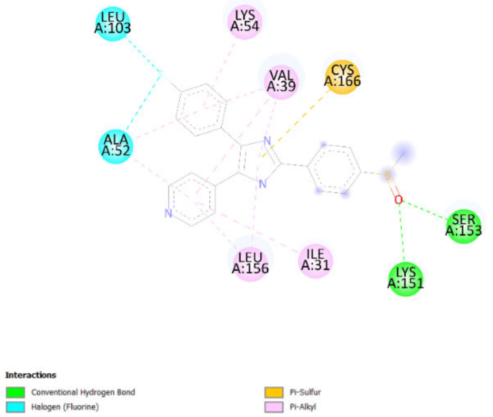

Chlorpyrifos oxon

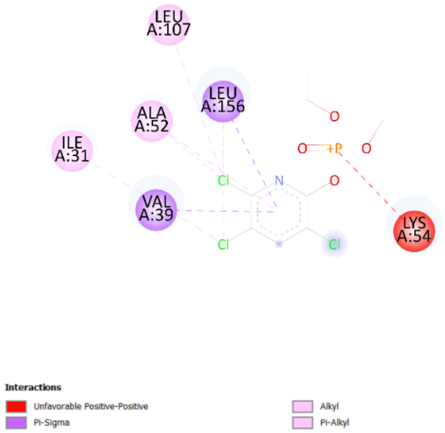

EGFR

AQ4*

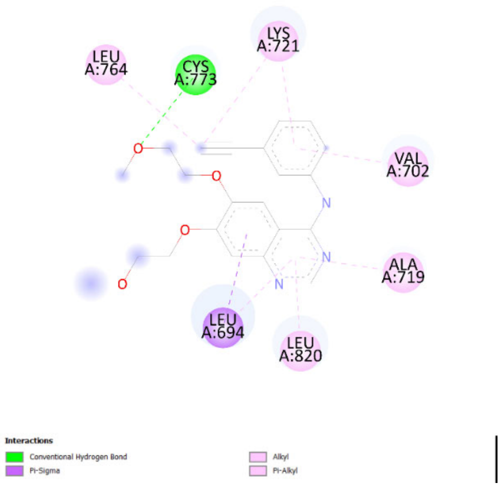

Diazinon oxon

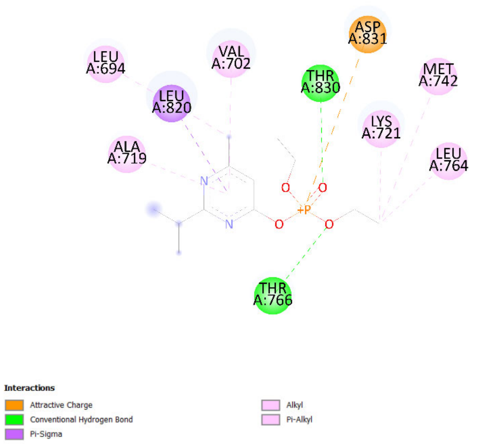

Chlorpyrifos oxon

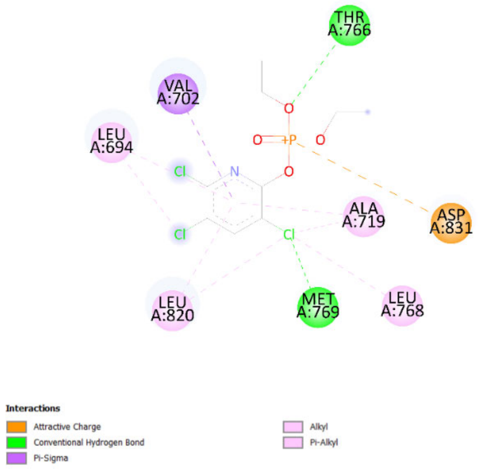

Paraoxon

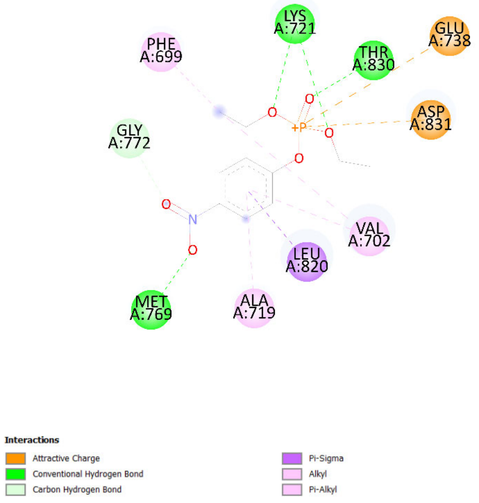

APH1A

PC1*

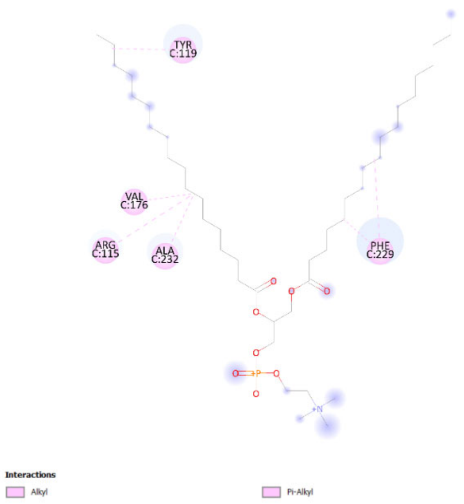

Diazinon oxon

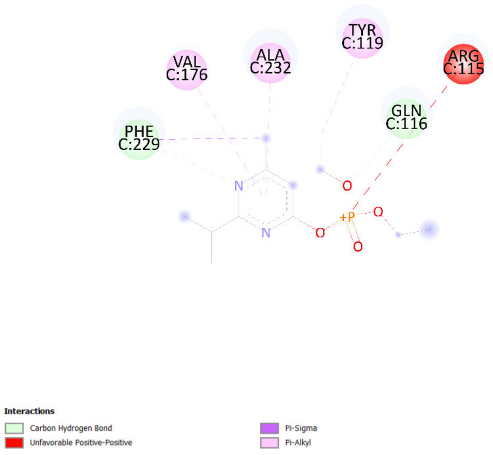

Paraoxon

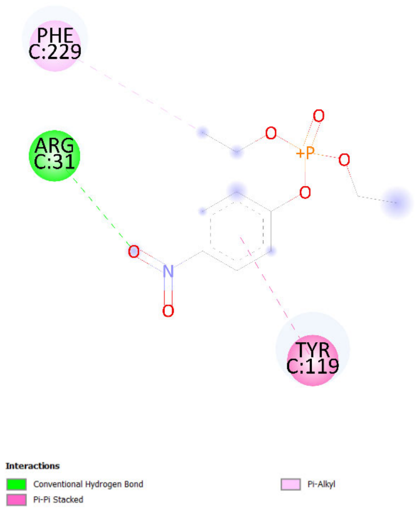

PTPN11

B2B*

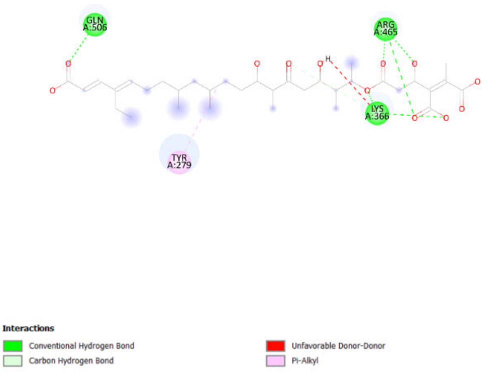

Diazinon oxon

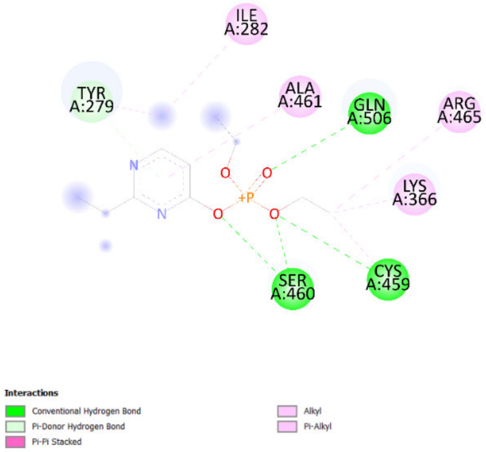

Paraoxon

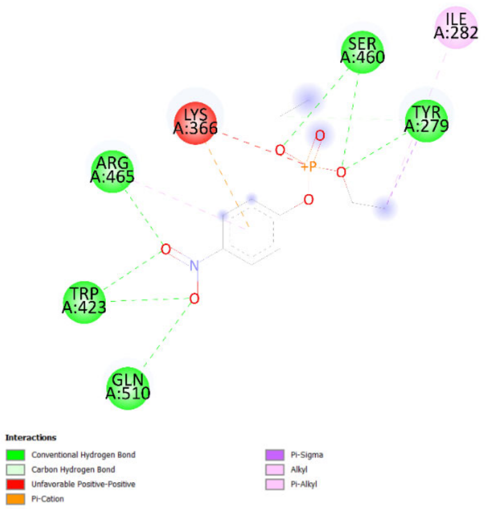

CALM1

TFP* Chlorpyrifos oxom

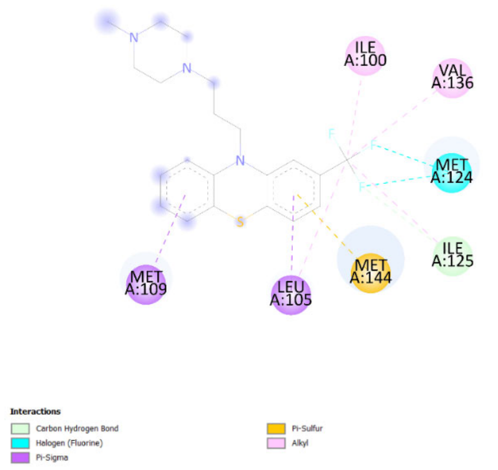

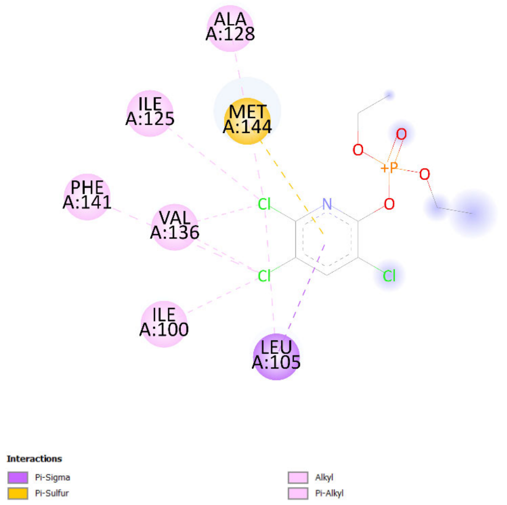

STAT3

KQV*

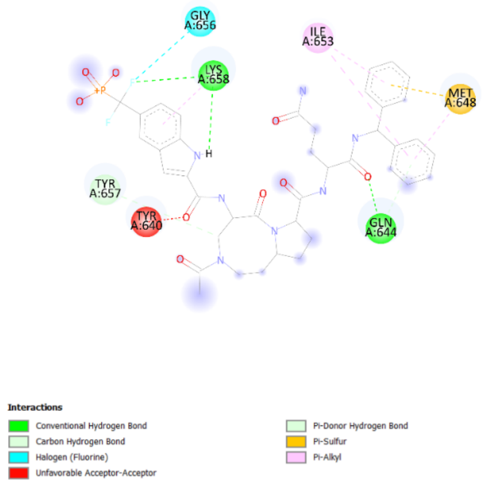

Diazinon oxon

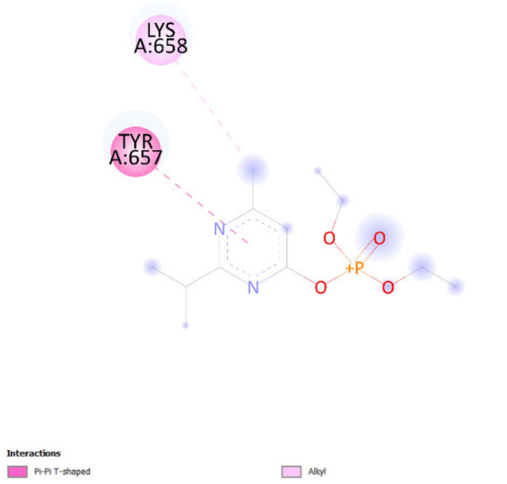

ERBB2

NAG*

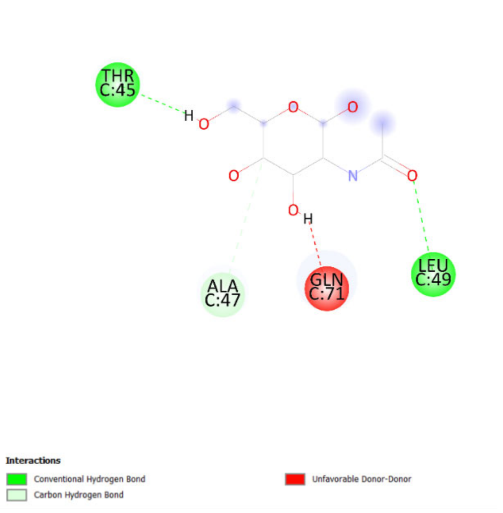

Paraoxon

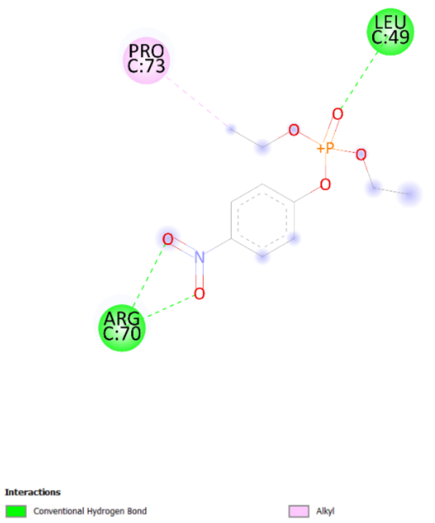

SRC

903*

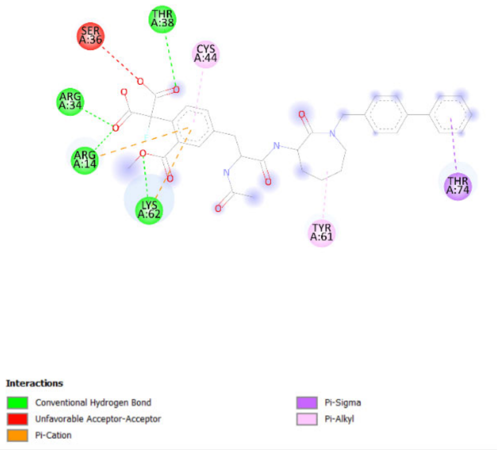

Diazinon oxon

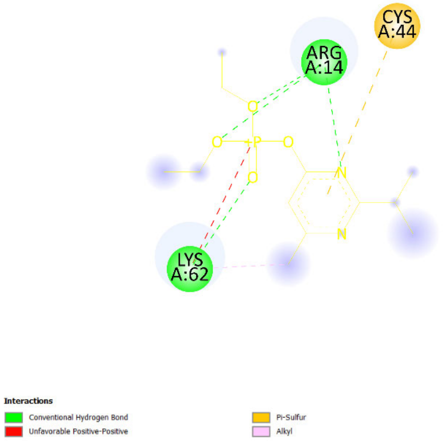

Chlorpyrifos oxon

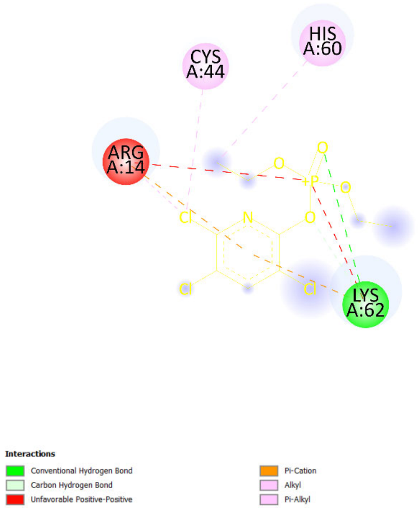

Paraoxon

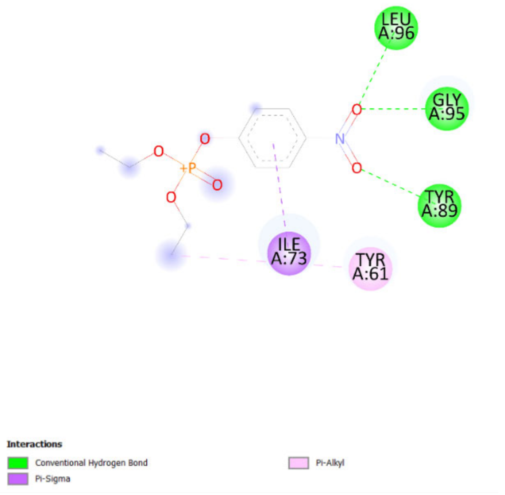

NCSTN

NAG*

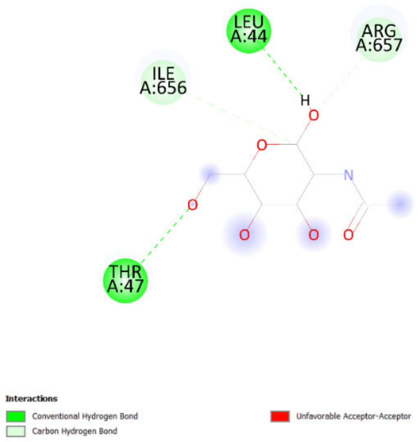

Diazinon oxon

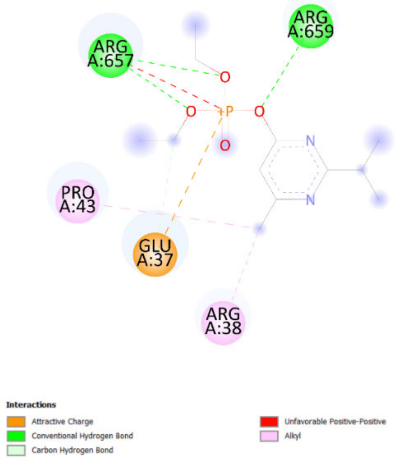

Paraoxon

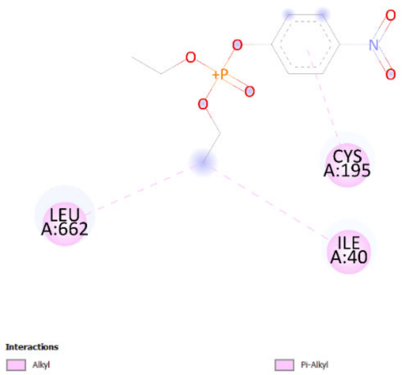

PSENEN

NAG*

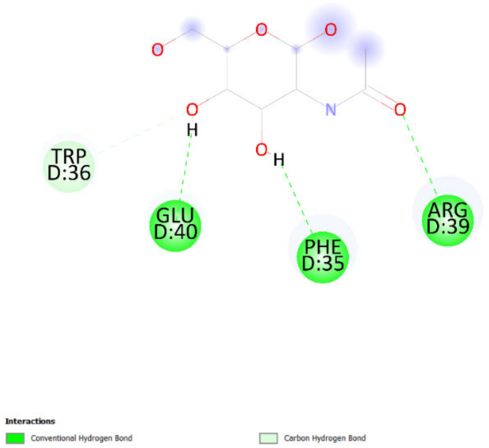

Diazinon oxon

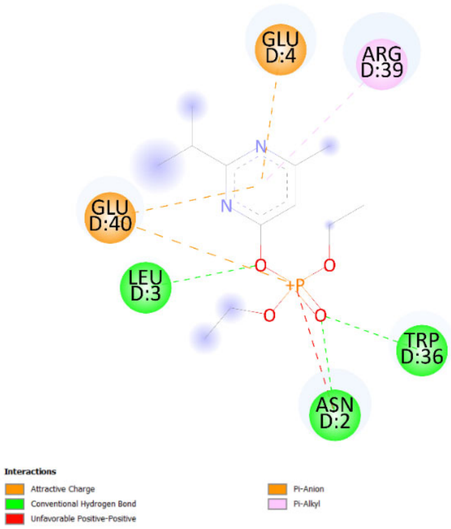

Paraoxon

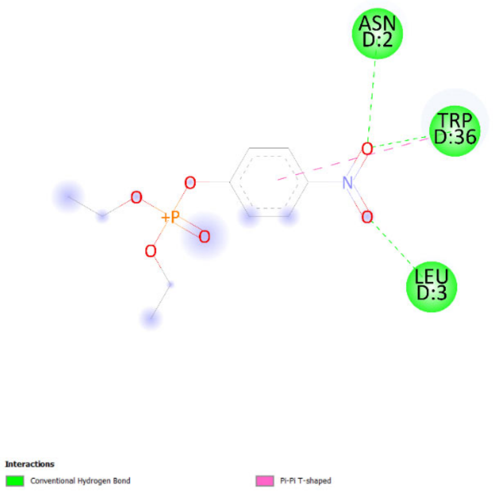

PTPN1

761*

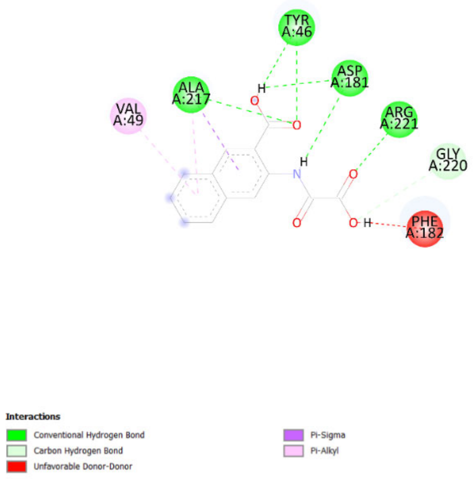

Chlorpyrifos oxon

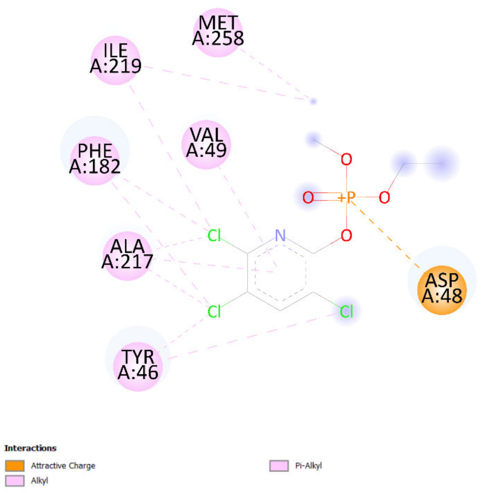

* original ligand
